# Multifunctional SnO_2_/Perovskite Interface Engineering for Efficient Perovskite Solar Cells

**DOI:** 10.1002/advs.202514595

**Published:** 2025-09-26

**Authors:** Keqing Huang, Wei Wang, Anh Dinh Bui, Wenzhong Ji, Felipe Kremer, Zhongshu Yang, Gabriel Bartholazzi, Yang Yu, Olivier Lee Cheong Lem, Bingchen He, Zhenhuang Su, Viqar Ahmad, Lichun Chang, Dang‐Thuan Nguyen, Yun Liu, Xingyu Gao, Junliang Yang, Kylie R. Catchpole, Heping Shen, Klaus J. Weber, The Duong

**Affiliations:** ^1^ School of Engineering The Australian National University Canberra Australian Capital Territory 2601 Australia; ^2^ Research School of Chemistry The Australian National University Canberra Australian Capital Territory 2601 Australia; ^3^ Centre for Advanced Microscopy The Australian National University Canberra Australian Capital Territory 2600 Australia; ^4^ Research School of Physics The Australian National University Canberra Australian Capital Territory 2600 Australia; ^5^ Australian National Fabrication Facility Research School of Physics The Australian National University Canberra Australian Capital Territory 2600 Australia; ^6^ Shanghai Synchrotron Radiation Facility (SSRF) Shanghai Advanced Research Institute Chinese Academy of Sciences Shanghai 201204 P. R. China; ^7^ Hunan Key Laboratory for Super‐microstructure and Ultrafast Process School of Physics Central South University Changsha 410083 P. R. China

**Keywords:** aluminum chloride, hydrolysis reaction, interface, perovskite solar cells, SnO_2_

## Abstract

Perovskite solar cells (PSCs) have shown significant advancements and commercial potential; however, their efficiency is often limited by defects in the bulk material and surface. Stability issues, such as ion migration and degradation of perovskite materials, further exacerbate this challenge. In this study, a strategy using aluminum chloride is introduced to eliminate hydroxyl groups and potassium ions from the tin dioxide (SnO_2_) surface, effectively reducing deprotonation of perovskite. This process also forms an ultra‐thin aluminum oxide layer at the SnO_2_/perovskite interface, functioning as a passivation layer. This modification decreases leakage current and charge carrier recombination, lowering the energy barrier for electron transport, resulting in enhanced open‐circuit voltage and overall efficiency. The approach achieved a certified efficiency of 26.29% in single‐junction n‐i‐p PSCs, marking the highest reported efficiency for the n‐i‐p PSCs utilizing SnO_2_ electron transport material. The devices retained 94% of their initial efficiency after 10 044 h in dry air (5% relative humidity) and demonstrated a *T*_80_ lifetime of over 500 h under continuous illumination, demonstrating superior stability compared to control cells. This research provides critical insights into engineering the chemical and physical interface properties and enhancing the photovoltaic performance of PSCs.

## Graphical abstract

Mobile ions and hydroxyl groups are eliminated from the surface of tin oxide layers by utilizing the hydrolysis of aluminum chloride. Meanwhile, an aluminum oxide passivation layer is constructed during the annealing process via the chemical reaction related to the resultant aluminum complexes. This strategy results in lower leakage current and better energy level alignment between tin oxide and perovskite. Accordingly, a certified efficiency of 26.29% and better device stability have been obtained with n‐i‐p structure.

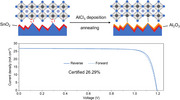

## 1 Introduction

The power conversion efficiency (PCE) of single‐junction perovskite solar cells (PSCs) has reached a certified 27.0% after over a decade of intensive research and development.^[^ ^1^ ^]^ This achievement can be largely attributed to the exceptional opto‐electrical properties inherent to perovskite materials and the solution processibility of PSCs, which facilitate the swift optimization of PSC performance through various methodologies, including but not limited to perovskite composition engineering,^[^ ^2^, ^3^, ^4^ ^]^ alterations in fabrication techniques,^[^ ^5^ ^]^ and the incorporation of novel and efficient charge transport materials.^[^ ^6^, ^7^, ^8^, ^9^ ^]^ Furthermore, interface modification is critical for enhancing both the performance and stability of PSCs. 2D perovskites or organic halides are generally employed to passivate the surface defects in perovskite films.^[^ ^10^, ^11^, ^12^, ^13^ ^]^ When it comes to the underlying perovskite interface, a range of materials were utilized to modulate the trap‐state density of the perovskite, improve charge carrier extraction, and alleviate residual stress within the perovskite films.^[^ ^14^, ^15^, ^16^, ^17^ ^]^ Given the dependence of the perovskite growth on the substrate characteristics, the pre‐deposition of specific materials has proven effective in improving the morphology and grain size of perovskite films.^[^ ^18^, ^19^, ^20^ ^]^ The aforementioned strategies contribute significantly to the enhancement of both efficiency and stability of PSCs.

Nowadays, several advanced cell configurations related to perovskites are under investigation, notably n‐i‐p (regular) PSCs utilizing tin dioxide (SnO_2_) as the electron transport layer,^[^ ^21^, ^22^ ^]^ p‐i‐n (inverted) PSCs employing a self‐assembled monolayer as the hole transport layer,^[^ ^9^ ^]^ and perovskite/silicon tandem solar cells.^[^ ^23^ ^]^ These technologies exhibit remarkable efficiencies and stability, highlighting their promising prospects for commercialization. Nonetheless, the operational instability of PSCs when exposed to sunlight remains a significant concern within the field. The instability is intrinsically linked to ion migration within the cells and chemical reactions induced by light and elevated temperature.^[^ ^24^, ^25^, ^26^ ^]^ Specifically, various ions including iodine ions and iodine interstitials in perovskite films are prone to migrate and ultimately accumulate at the interfaces, which negatively affects the interface quality and the energy level alignment between the perovskite and charge transport layers.^[^ ^27^ ^]^ Furthermore, mobile ions such as lithium ions and silver ions originating from adjacent layers can also contribute to the degradation of device performance.^[^ ^24^, ^27^ ^]^ Chemical reactions occurring within the cells represent an additional challenge; for instance, the decomposition and deprotonation of perovskites can be triggered by ultraviolet light and hydroxyl groups on the surface of metal oxides, respectively.^[^ ^28^, ^29^, ^30^, ^31^, ^32^ ^]^ The presence of residual lead iodide (PbI_2_) in perovskite films has been confirmed to decompose into metallic lead impurities upon light exposure, leading to deep‐level defect states in the perovskite and degradation of device performance during operation.^[^ ^25^, ^26^ ^]^ In contrast to the instability of perovskites exacerbated by humidity and oxygen, these issues cannot be mitigated through encapsulation strategies. Therefore, it is imperative to develop methods to eliminate mobile ions within PSCs and inhibit deleterious chemical reactions associated with perovskite materials.

Herein, we present a strategy aimed at mitigating the presence of mobile ions and hydroxyl groups on the surface of SnO_2_. Specifically, aluminum chloride (AlCl_3_) is introduced at the SnO_2_/perovskite interface. The hydrolysis of AlCl_3_ occurs and subsequently reacts with potassium hydroxide (KOH) that is utilized to stabilize the SnO_2_ precursor in aqueous colloidal dispersion. Results obtained from X‐ray photoelectron spectroscopy (XPS) and time‐of‐flight secondary ion mass spectrometry (ToF‐SIMS) indicate that K⁺ ions on the SnO_2_ surface are effectively eliminated through the neutralization reaction and the additional aqueous‐based spin coating process. The resultant product following hydrolysis and subsequent annealing is identified as aluminum oxide (Al_2_O_3_). Characterization studies reveal that the Al_2_O_3_ layer significantly reduces leakage current caused by rough substrates and passivates defects located at the bottom of perovskite films. Additionally, improved energy level alignment is achieved between SnO_2_ and perovskite. Consequently, the performance of single‐junction n‐i‐p PSCs based on this modified SnO_2_/perovskite interface shows notable improvement, achieving a certified efficiency of 26.29%. Moreover, the target cells demonstrate superior stability under both illumination and dry air conditions compared to control cells. This work proposes a straightforward method to engineer the properties of the perovskite interface, significantly suppressing the degradation of PSCs.

## 2 Results and Discussion

### 2.1 Impact of Aluminum Chloride on the Substrate

**Figure 1:**
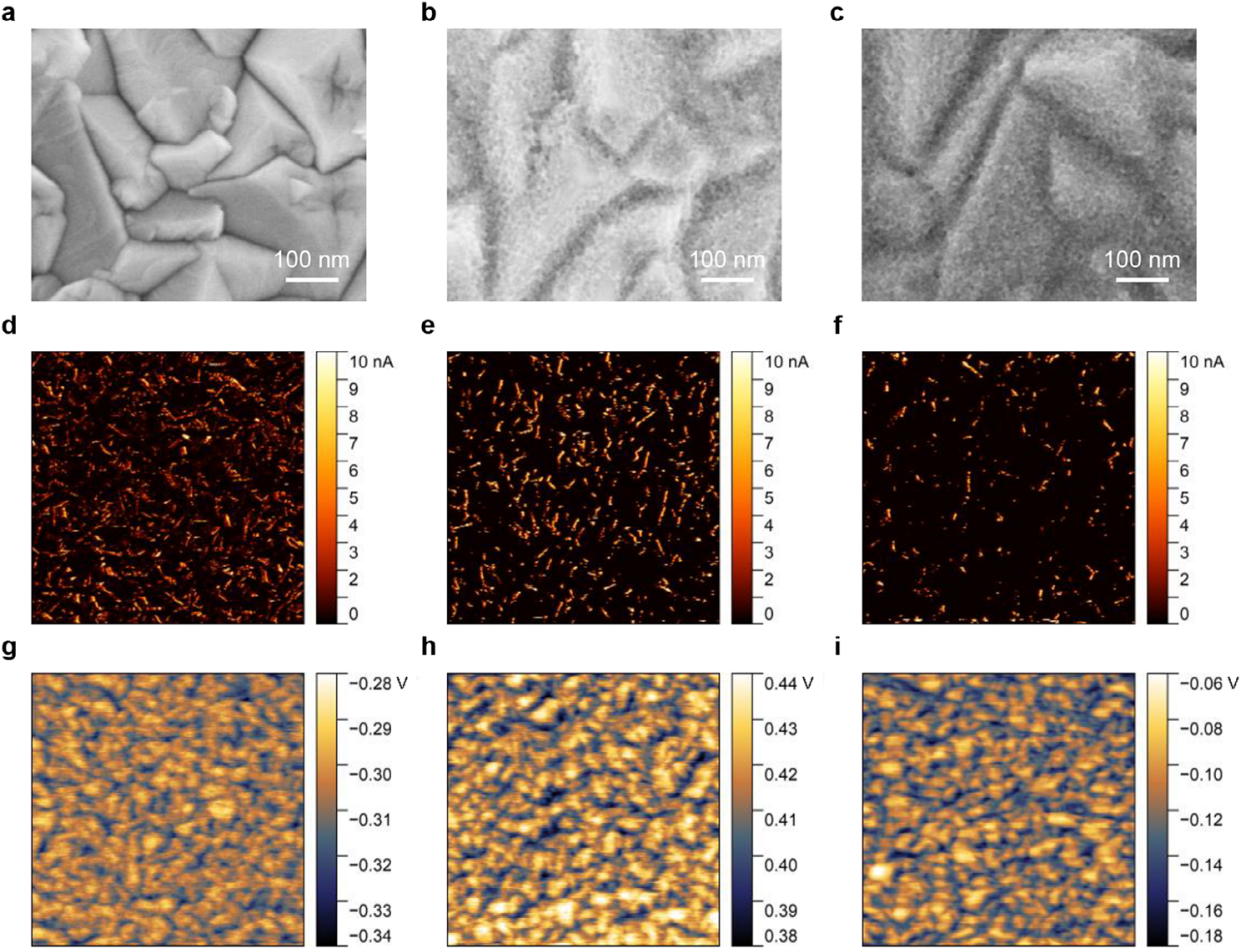
SEM images of a) FTO, b) FTO/SnO_2_, and c) FTO/SnO_2_/AlCl_3_; c‐AFM images (5 × 5 µm) of d) FTO, e) FTO/SnO_2_, and f) FTO/SnO_2_/AlCl_3_; KPFM images (5 × 5 µm) of g) FTO, h) FTO/SnO_2_, and i) FTO/SnO_2_/AlCl_3_.

In accordance with the proposed strategy, AlCl_3_ solution was spin‐coated onto a Glass/FTO/SnO_2_ substrate [FTO: fluorine‐doped tin oxide] to modify the electrical and chemical properties of the SnO_2_ layer. The samples without and with AlCl_3_ are denoted as control and target, respectively. The surface morphology of the substrate exhibited minimal changes after SnO_2_ deposition (**Figure** **1**a,b), preserving the characteristics of the FTO substrate with only slight texture alterations. Notably, the FTO crystal peaks remained visible beneath the SnO_2_ layer (Figure 1b). This indicates that the thin SnO_2_ layer is insufficient to uniformly and completely cover the FTO substrate, which typically has a high surface roughness of ≈30 nm (Figure S1a, Supporting Information). In contrast, most peaks of the FTO were covered after the deposition of AlCl_3_ (Figure 1c), suggesting enhanced substrate coverage. It should be attributed to the additional AlCl_3_ film serving as a capping layer for most of the exposed FTO peaks.

Atomic force microscopy (AFM) height images showed a decrease in root‐mean‐square roughness of the substrate from 36.6 to 27.4 nm after SnO_2_ deposition (Figure S1, Supporting Information). After the introduction of AlCl_3_, the target sample exhibited a surface roughness of 29.2 nm, showing negligible difference compared to that of the control sample. The current intensity obtained from the conductive atomic‐force microscopy (c‐AFM) images correlates with FTO surface morphology, with higher currents observed at peaks (Figure 1d). After the deposition of SnO_2_, the average current remains almost identical (0.581–0.519 nA), but the extent of bright areas decreased, suggesting that most of the substrate area was covered by SnO_2_ (Figure 1e). It is necessary to point out that numerous peaks remain exposed, as high currents are still observed at these peaks. In contrast, the bright areas significantly diminished after the introduction of AlCl_3_, resulting in a substantial decrease in the average current to 0.283 nA (Figure 1f). These results are consistent with the scanning electron microscopy (SEM) images aforementioned, suggesting that the AlCl_3_ layer enhances the coverage over the substrate. The surface potentials of the Glass/FTO, Glass/FTO/SnO_2_, and Glass/FTO/SnO_2_/AlCl_3_ samples were measured as −0.308, 0.415, and −0.119 V, respectively (Figure 1g–i), indicating a change in the work function of the substrates due to AlCl_3_ modification. Kelvin Probe measurements revealed work functions of 4.61, 4.28, and 4.48 eV for the respective samples (Figure S2, Supporting Information). The change in work function may enhance electron extraction and transport by alleviating the energy barrier between SnO_2_ and perovskite.^[^ ^16^, ^18^, ^33^ ^]^ It is worth noting that the AlCl_3_ layer does not negatively impact substrate transmittance, ensuring effective light absorption by the perovskite absorber; on the contrary, a slight enhancement is observed in the short‐wavelength region (Figure S3, Supporting Information).

**Figure 2:**
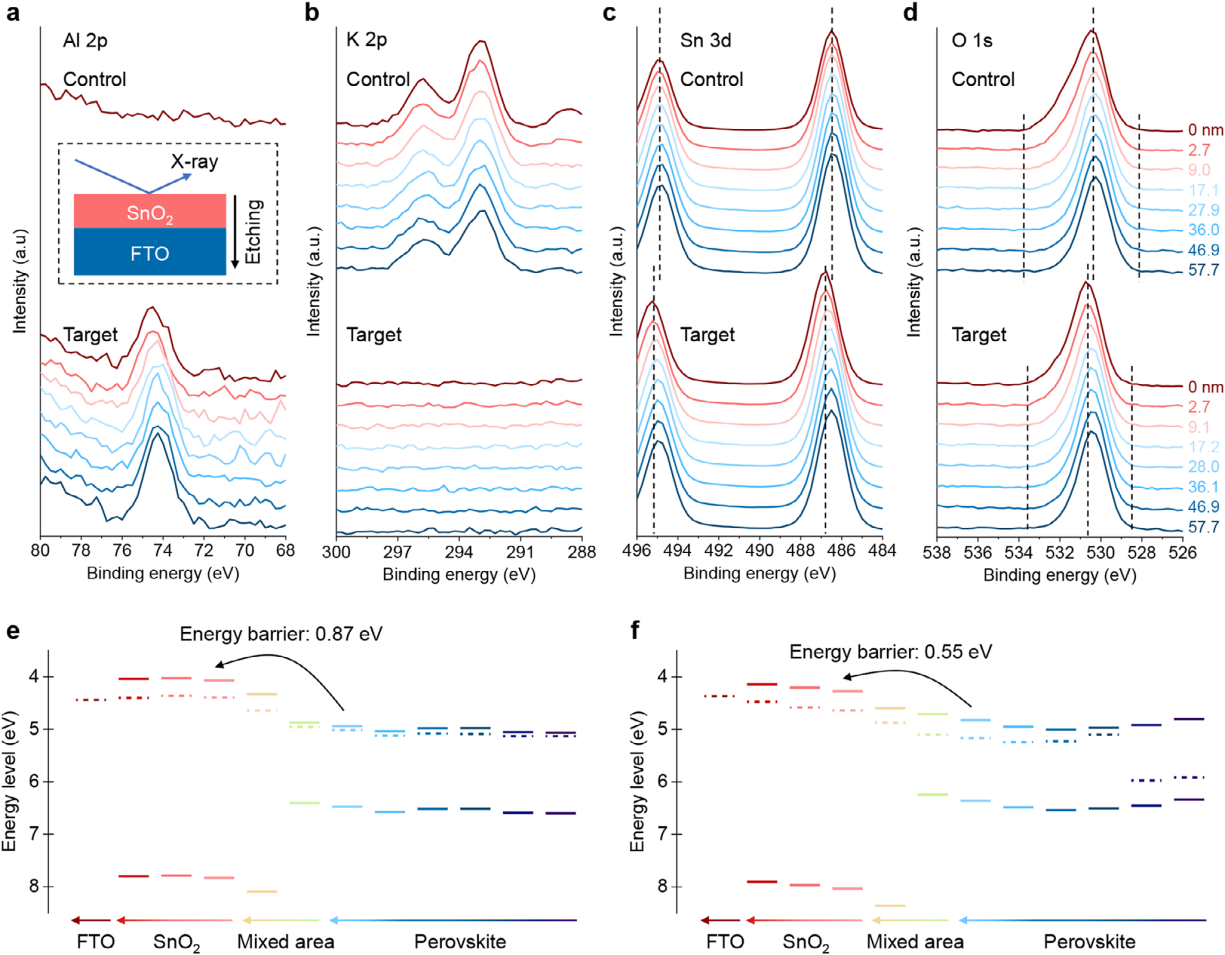
Depth‐dependent XPS spectra of a) Al 2p, b) K 2p, c) Sn 3d, and d) O 1s of control and target samples; energy level alignment of e) control and f) target samples with perovskite films.

Depth‐dependent XPS was employed to investigate the impact of AlCl_3_ on the elements and functional groups of Glass/FTO/SnO_2_. As illustrated in Figure S4 (Supporting Information), the elements oxygen (O), tin (Sn), and fluorine (F) exhibited negligible differences between the control and target samples in terms of atomic ratio and element distribution in the profiles. The presence of the potassium (K) in the control sample is attributed to the KOH in the SnO_2_ colloidal dispersion, which is used to stabilize the SnO_2_ nanoparticles.^[^ ^34^ ^]^ As expected, aluminum (Al) was only found in the target sample (**Figure** **2**a). However, it is interesting to observe that the K element was not detected in the target sample (Figure 2b). Apart from that, the carbon signal intensity was found to be reduced on the surface of the target sample (Figure S5a, Supporting Information). Despite the introduction of AlCl_3_ on the SnO_2_ layer, chlorine (Cl) was not detected in the target sample (Figure S4b, Supporting Information). These surprising results will be discussed in more detail later. Furthermore, noticeable shifts toward higher binding energy in the XPS peaks of Sn 3d5 (from 486.5 to 486.8 eV) and O 1s (from 530.4 to 530.7 eV) were observed after the deposition of AlCl_3_ (Figure 2c,d). These shifts may be attributed to bond formation between the Al and O elements, or to trapped charges induced by the resultant compound.^[^ ^35^ ^]^ Importantly, Figure 2d demonstrates that the width of the O 1s peak and the intensity of O 1s satellite peak are reduced by AlCl_3_. The satellite peak generally corresponds to hydroxyl groups or oxygen vacancies in SnO_2_ layers. This result indicates that the incorporation of AlCl_3_ significantly reduces the hydroxyl groups and/or oxygen vacancies in SnO_2_ layers. Signals corresponding to N 1s and F 1s show negligible differences between the control and target samples, likely due to the low concentration of F in the FTO substrate and mild contamination from nitrogen (N_2_) in ambient air. Further details are available in the depth‐dependent XPS spectra (Figure S5, Supporting Information) and the full XPS spectra for both control and target samples (Figure S6, Supporting Information).

The influence of AlCl_3_ on the energy levels of SnO_2_ is investigated using depth‐dependent ultraviolet photoelectron spectroscopy (UPS). It is noted that the surface of the sample can be contaminated by CO_2_ and O_2_ from air, as confirmed by XPS spectra and our previous result.^[^ ^18^ ^]^ Thus, the analysis of UPS spectra starts at a depth of 4.8 nm beneath the surface. The results show that the conduction band of SnO_2_ at the depth of 4.8 nm increases from 3.92 to 4.10 eV after the modification with AlCl_3_ (Figure S7, Supporting Information). As the etching depth increases to 30 nm, the conduction band for both the control and target samples converges to 4.02 eV (Table S1, Supporting Information). This suggests that the surface of the SnO_2_ layer is more readily modified by the AlCl_3_ coating compared to the bulk of SnO_2_.

Given that the energy level alignment between SnO_2_ and perovskite plays a vital role in charge carrier extraction and transport, the samples with the structure of Glass/FTO/SnO_2_/perovskite are investigated by depth‐dependent UPS as well. As shown in Figure 2e, an energy barrier of 0.87 eV is measured at the SnO_2_/perovskite interface in the control sample, which hinders electron extraction at the interface.^[^ ^16^ ^]^ In the target sample, the energy barrier is reduced to 0.55 eV (see more details in Figure S8 and Table S2, Supporting Information). It can be attributed to the influence of AlCl_3_ on the energy level of the SnO_2_ surface, and the change is consistent with the results of Kelvin probe force microscopy (KPFM) and Kelvin Probe aforementioned. Our approach also causes significant shifts in the energy levels of the perovskite. The conduction band at the etching depth of 707 nm shifts from 4.94 to 4.82 eV, while the work function changes from 5.02 to 5.17 eV (Figure 2e,f). This means the perovskite has transformed from heavily n‐type to weakly n‐type. Apart from that, the perovskite at the etching depth of 11 nm transforms from n‐type to weakly p‐type after the modification of AlCl_3_, with the valence band shifting from 6.59 to 6.45 eV. These alterations are expected to improve energy level alignment with the hole transport layer, facilitating hole extraction. The changes in energy levels of the perovskite surface are likely attributed to the elimination of potassium and the reduction of the white phase (namely PbI_2_ or (PbI_2_)_2_RbCl) at the surface of perovskite films, which will be demonstrated later in the SEM images. Kelvin probe measurements (Figure S9, Supporting Information) show that while the valence band maximum (VBM) of 2,2′,7,7′‐tetrakis(*N*,*N*‐dipmethoxyphenylamine)‐9,9′‐spirobifluorene (spiro‐OMeTAD) remains at 4.96 eV, the VBM of the perovskite slightly shifts upward after AlCl_3_ incorporation, which is consistent with UPS results. It reduces the energy loss for holes at the perovskite/spiro‐OMeTAD interface and is beneficial to cell performance.

### 2.2 Element Distribution in PSCs and Improved Perovskite Quality

**Figure 3:**
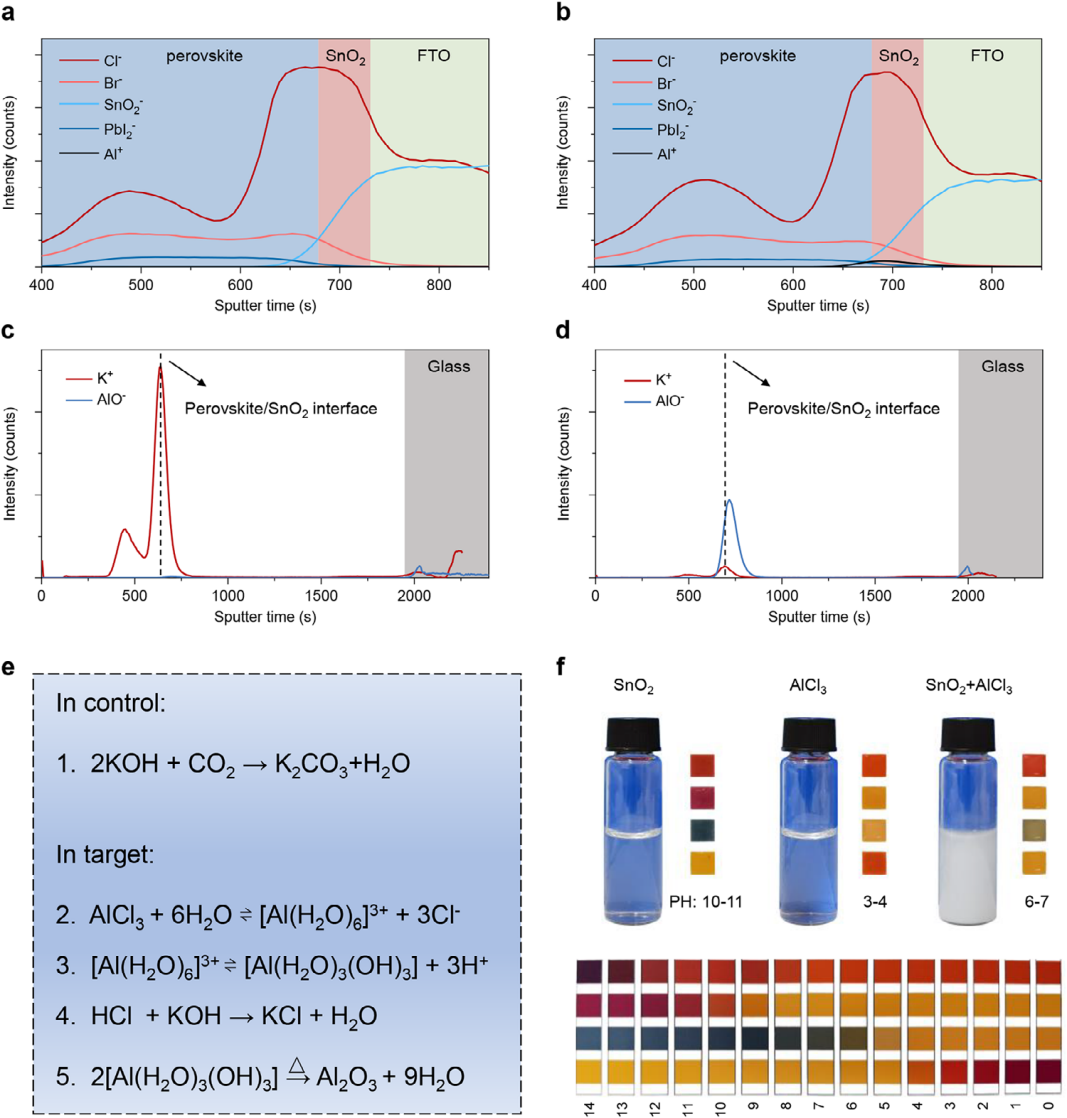
ToF‐SIMS results of a) control and b) target PSCs; K and Al element distribution of c) control and d) target PSCs; e) proposed scheme of chemical reactions of control and target samples; f) photographs of SnO_2_ precursor (3.17% in H_2_O colloidal dispersion), AlCl_3_ solution (2 mg mL^−^^1^ in H_2_O), the solution mixing with 3.17% SnO_2_ and 2 mg mL^−^^1^ AlCl_3_, and pH card.

ToF‐SIMS was utilized to examine the element distribution in the PSCs. The results show that the distributions of Br^−^, SnO_2_^−^, and PbI_2_^−^ do not have obvious variations before and after the deposition of AlCl_3_ (**Figure** **3**a,b). In contrast, the intensity of Al⁺ signal significantly increases at the SnO_2_/perovskite interface in the target sample. Although AlCl_3_ is deposited on the SnO_2_ surface, the changes in the Cl^−^ signal intensity are negligible. The Cl^−^ signal presents two distinct peaks in both TOF‐SIMS results, which are located at the top surface of the perovskite film and at the SnO_2_/perovskite interface, respectively (Figure 3a,b). The peak identified at the top surface of the perovskite can be attributed to (PbI_2_)_2_RbCl, a reaction product of PbI_2_ and RbCl, which serves to effectively stabilize the perovskite phase.^[^ ^36^ ^]^ The other peak is ascribed to the robust bonding between Cl^−^ in the perovskite and Sn^4+^ in SnO_2_. Despite the evaporation of Cl during the annealing process of the perovskite films, this bonding facilitates the attraction and retention of Cl^−^ ions at the SnO_2_/perovskite interface. Previous reports have also confirmed numerous Cl^−^ ions at this interface, when methylammonium chloride is used as an additive in perovskite precursor.^[^ ^18^, ^19^ ^]^ Additionally, a weak signal corresponding to Al element is identified in the control sample, likely stemming from the Al present in the glass substrate (Figure 3c,d). The Rb element is predominantly located at the surface of the perovskite film and exhibits a tendency to migrate into the spiro‐OMeTAD layer, while Li^+^ ions are found to migrate into the perovskite layers (Figure S10, Supporting Information). A previous report showed a similar phenomenon regarding Li^+^ ion migration.^[^ ^24^ ^]^ This indicates that both Rb^+^ and Li^+^ ions are prone to migrate within the cells. A distinct peak of K element was detected at the perovskite surface of control sample, suggesting that K^+^ ions exhibit similar nature to Rb^+^ ions and Li^+^ ions, namely easy migration in devices. The intensity of the K element signal at both the perovskite surface and the SnO_2_/perovskite interface is remarkably reduced in the target sample, suggesting that the AlCl_3_ coating effectively eliminates these mobile K^+^ ions. The result is consistent with the depth‐dependent XPS spectra above, and the trace amounts of K^+^ ions in the target sample should be ascribed to the SnO_2_ layer or the glass substrate (Figure 3c,d).

Based on these observations, we propose the reaction mechanisms illustrated in Figure 3e to elucidate the intriguing phenomena discussed above. In the control sample, the presence of a considerable amount of KOH leads to a chemical reaction between KOH and CO_2_ in ambient air (reaction 1 of Figure 3e), accounting for the strong intensity of the carbon element observed in XPS (Figure S5a, Supporting Information). However, for the target sample, a reversible hydrolysis reaction occurs between Al^3+^ ions and H_2_O (reaction 2) when AlCl_3_ is dissolved in water, leading to the formation of [Al(H_2_O)_6_]^3+^ complexes and Cl^−^ anions. Given the highly positive charges of Al^3+^ in [Al(H_2_O)_6_]^3+^ complexes, some hydrogen atoms are displaced from the complexes, thus producing H^+^ (reaction 3). These two reactions generate considerable heat and result in an acidic solution (Figure 3f). When this solution is spin‐coated onto the SnO_2_ film, H^+^ cations react with the KOH present in the SnO_2_ film to generate water and K^+^ cations (reaction 4). Given the high solubility of K^+^ and Cl^−^ in aqueous solutions, these two elements are washed away during the spin‐coating process. This accounts for their absence in the target sample (Figure 2b; Figure S4b, Supporting Information). Besides, reaction 4 explains the observed reduction in hydroxyl groups in the target samples (Figure 2d). Chemical reactions 3 and 4 can be further corroborated by the agglomeration of SnO_2_ nanoparticles and the change in pH of the solution when AlCl_3_ is mixed with SnO_2_ colloid dispersion (Figure 3f). In the SnO_2_ dispersion, numerous hydroxyl groups are attached to the surface of SnO_2_ nanoparticles, inhibiting the attractive interactions between these SnO_2_ nanoparticles. When these hydroxyl groups are consumed by AlCl_3_, the pH of the solution decreases and the interactions between SnO_2_ nanoparticles are significantly enhanced, resulting in their agglomeration. In addition, it is assumed that reaction 5 occurs during the annealing of the AlCl_3_ layer, resulting in the formation of Al_2_O_3_, which has been confirmed to passivate defects at the SnO_2_/perovskite interface.^[^ ^35^, ^37^ ^]^ The presence of AlO^−^ fragments, as observed in the ToF‐SIMS results for the target sample (Figure 3d), further confirms the existence of Al_2_O_3_. In summary, Figure 3e provides a comprehensive theoretical framework for the findings observed in XPS and ToF‐SIMS. It indicates that AlCl_3_ serves not only as an interface modifier but also greatly changes the properties of the SnO_2_ film, including substrate coverage, surface potential, and functional groups.

Grazing incidence X‐ray diffraction (GIXRD) patterns of silicon/SnO_2_ and silicon/SnO_2_/AlCl_3_ samples suggest that the deposition of AlCl_3_ does not introduce additional diffraction peaks for the SnO_2_ layer (Figure S11, Supporting Information). We hypothesize that the introduced layer is too thin to be detected by GIXRD. However, transmission electron microscopy (TEM) cross‐section images reveal an extra layer on the surface of SnO_2_ in the target sample, which displays clear fringe patterns (Figure S12, Supporting Information). A lattice spacing of ≈0.33 nm is observed in SnO_2_ nanoparticles, which corresponds to the (110) plane of tetragonal SnO_2_.^[^ ^38^ ^]^ Meanwhile, the additional layer exhibits a lattice spacing of ≈0.35 nm, which can be attributed to the (102) plane of α‐Al_2_O_3_ (corundum structure).^[^ ^39^ ^]^ It is necessary to note that this layer exists as a discontinuous layer and exhibits non‐uniform thickness across the substrate, similar to the SnO_2_ layer (Figure S13, Supporting Information). This observation is consistent with the c‐AFM results. We speculate that it serves as a tunnelling/passivation layer rather than functioning as a dense dielectric barrier. A schematic of the control and target SnO_2_/perovskite interface is depicted in Figure S14 (Supporting Information). It is assumed that the Al_2_O_3_ layer can interact with undercoordinated Pb^2+^ ions at the SnO_2_/perovskite interface and prevent direct contact between FTO peaks and perovskite, which is beneficial to the suppression of non‐radiative recombination and reduction of leakage current.

**Figure 4:**
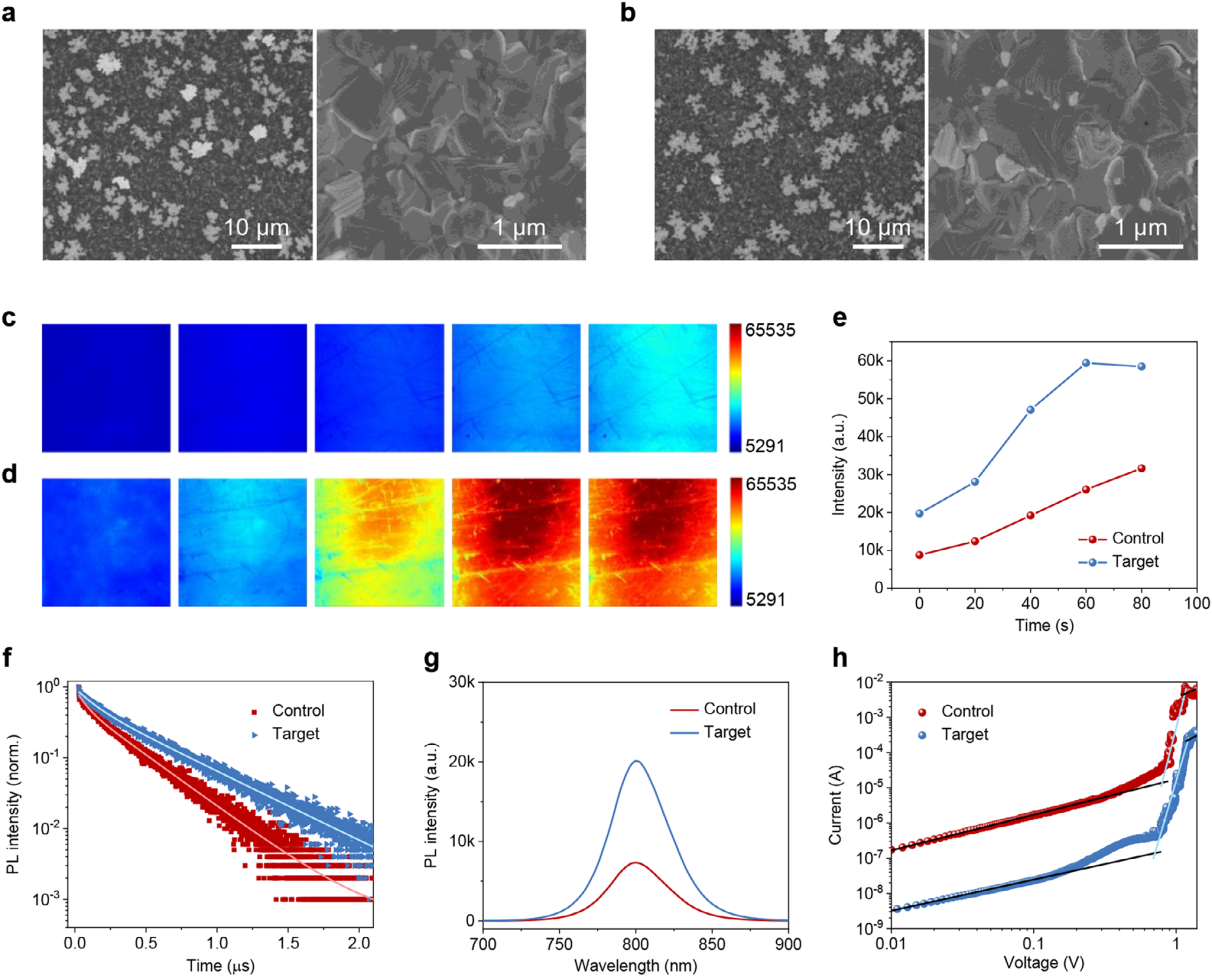
SEM images of a) control and b) target perovskite films; PL images of c) control and d) target PSCs under light soaking; e) PL intensity of control and target PSCs under light soaking; f) time‐resolved PL spectra of control and target perovskite films; g) steady PL spectra of control and target perovskite films; h) current–voltage curves of the electron‐only devices measured in dark.

We then conducted the SEM to investigate the perovskite film morphology in order to understand the impact of the substrate on the growth of perovskites. The result shows that both control and target perovskite films exhibit many white phases at their surface (**Figure** **4**a,b), which are associated with PbI_2_ and (PbI_2_)_2_RbCl.^[^ ^33^, ^36^, ^40^ ^]^ However, the white phases are slightly reduced in target samples. In detailed images, both perovskite films present similar morphologies (Figure 4a,b). The perovskite grain size is slightly increased after the introduction of AlCl_3_ layer, which may account for the reduction of white phases at the surface as large perovskite grains are less likely to decompose into PbI_2_ during annealing process.^[^ ^18^, ^40^ ^]^ The perovskite grains in both samples are sufficiently large to span the entire perovskite film (Figure S15, Supporting Information), so charge carriers can travel through the perovskite film without crossing grain boundaries, which would facilitate carrier transport and suppress non‐radiative recombination. Grazing incidence wide‐angle X‐ray scattering (GIWAXS) patterns reveal that both perovskite films exhibit similar crystal planes. However, the diffraction peak at the scattering wave vector *q* value of 9.77 nm^−1^ is enhanced in the target sample, which is associated with the (100) crystal plane of perovskite (Figure S16, Supporting Information). Apart from that, the diffraction peak corresponding to PbI_2_ is slightly weakened, which is consistent with SEM images. These results indicate that the inserted layer between perovskite and SnO_2_ improves the quality of perovskite films.

Photoluminescence (PL) images of control and target PSCs were measured to investigate the charge carrier recombination within the cells. Both cells show an increase in PL intensity when exposed to light (Figure 4c,d), which corresponds to the light soaking phenomenon observed prior to measuring the current density–voltage (*J–V*) curves of PSCs. Importantly, the target cell shows significantly higher PL intensity than the control cell across all observations. The average PL intensity for both cells is presented in Figure 4e, indicating that the PL intensity of the target sample is approximately double that of the control sample. This suggests that non‐radiative recombination of charge carriers in the target cell is greatly suppressed. Time‐resolved PL spectra show that the carrier lifetime increases from 271.1 to 400.5 ns after the incorporation of AlCl_3_ (Figure 4f and Table S3, Supporting Information). Steady PL spectra further confirm that a higher PL intensity can be obtained in the target sample (Figure 4g). It is noted that the samples with a structure of Glass/FTO/SnO_2_/perovskite were utilized in time‐resolved PL spectra and steady PL spectra, and the incident light comes from the glass side. Therefore, the results are only affected by the perovskite films, SnO_2_ layers, and the SnO_2_/perovskite interface. According to the results from XPS and ToF‐SIMS, it is likely that carrier recombination at the SnO_2_/perovskite interface is significantly suppressed by the resultant compound, namely Al_2_O_3_. Space charge‐limited current (SCLC) was used to analyze the changes in trap‐state density (*N*_t_) of perovskite films. As shown in Figure 4h, the trap‐filled limit voltage (*V*_TFL_) for the control and target perovskite films is 0.803 and 0.709 V, respectively. *N*_t_ of perovskite films can be determined via Equation (1)

(1) $$N_{t}=\frac{2\varepsilon_{0}\varepsilon V_{\mathrm{TFL}}}{eL^{2}}$$

where *e* is the electron charge, *ε* is the relative dielectric constant of formamidinium lead triiodide (*ε* = 46.9),^[^ ^41^ ^]^ *ε*_0_ is the vacuum permittivity, and *L* is the thickness of the perovskite film (≈750 nm, as shown in Figure S15, Supporting Information). Accordingly, the *N*_t_ is found to decrease from 7.32 × 10^15^ to 6.46 × 10^15^ cm^−3^ after the introduction of AlCl_3_, demonstrating the passivation of defects at the SnO_2_/perovskite interface. When phenethylammonium iodide (PEAI) passivation layer for the perovskite surface was employed, the *V*_TFL_ for control and target perovskite was reduced to 0.436 and 0.341 V, respectively (Figure S17, Supporting Information). The *N*_t_ of control and target perovskite films was reduced to 3.97 × 10^15^ and 3.11 × 10^15^ cm^−3^, respectively. Even though these values are still much higher than that of a perovskite single crystal, they are falling within the reasonable range reported in the literature.^[^ ^42^, ^43^, ^44^ ^]^ More importantly, the results suggest that our strategy is effective in reducing the trap density of perovskite, irrespective of the properties of the perovskite surface.

### 2.3 PSCs Performance and Stability

**Figure 5:**
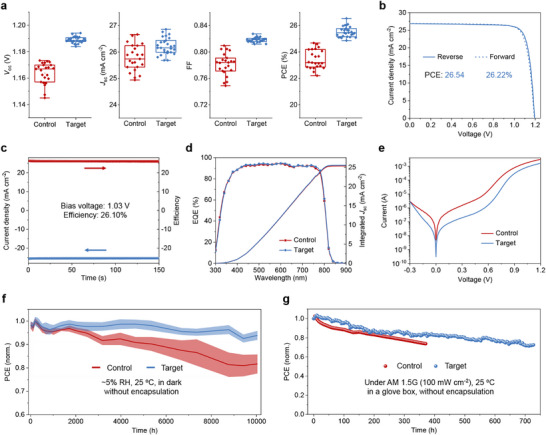
a) Photovoltaic parameters of control and target PSCs; b) *J–V* curves and c) steady‐state efficiency of champion target cell; d) EQE spectra of control and target PSCs; e) current–voltage curves of control and target PSCs under dark conditions; f) long‐term stability of the unencapsulated PSCs (six cells for each condition) stored in dry air (relative humidity of ≈5% and ≈25 °C); g) operational stability of the unencapsulated PSCs under one sun illumination (100 mW cm^−2^).

Both control and target PSCs with the structure of Glass/FTO/SnO_2_/perovskite/PEAI/spiro‐OMeTAD/Au were fabricated. **Figure** **5**a shows that the proposed strategy achieved open–circuit voltage (*V*_oc_) increasing from 1.164 to 1.189 V and fill factor (FF) rising from 0.781 to 0.819. The average short–circuit current density (*J*_sc_) showed slight growth from 25.78 to 26.21 mA cm^−2^. Consequently, the average PCE from reverse scanning increased from 23.43% to 25.51% after the incorporation of AlCl_3_. The *J–V* curves of PSCs with varying concentrations of AlCl_3_ are presented in Figure S18 (Supporting Information), showing that *V*_oc_ increases with higher AlCl_3_ concentrations. However, a slight decrease in FF was observed when the concentration of AlCl_3_ reached 4 mg mL^−^^1^ (Table S4, Supporting Information). To further elucidate the impact of AlCl_3_ concentration, the shunt resistance (*R*_sh_) and series resistance (*R*_s_) were extracted from reverse‐scanned *J–V* curves (Table S5, Supporting Information). *R*_sh_ increases significantly from 2574 to 4365 Ω cm^2^ when the concentration rises from 0 to 2 mg mL^−1^, consistent with improved defect passivation and reduced leakage pathways. At 4 mg mL^−1^, *R*_sh_ drops markedly to 2193 Ω cm^2^, correlating with the FF decrease and the reduced PCE. In contrast, *R*_s_ varies only slightly (0.45–0.30 Ω cm^2^), suggesting it plays a minor role in the performance trends. Figure 5b shows the *J–V* curves of the champion target cell with negligible hysteresis, which achieved an efficiency (*V*_oc_, *J*_sc_, and FF) of 26.54% (1.194 V, 26.86 mA cm^−2^, and 0.827) under reverse scan. The exceptionally high *J*_sc_ can be attributed to several key factors: the 100 nm magnesium fluoride (MgF_2_) anti‐reflection layer effectively reduces light reflection on the glass side; the rough FTO substrate helps minimize reflection at the SnO_2_/perovskite interface; the 750 nm‐thick perovskite layer ensures efficient absorption of incident light; and the 100 nm‐thick gold electrode enhances the reflection and subsequent reabsorption of light that passes through the perovskite layer. A steady‐state efficiency of 26.10% was obtained under AM 1.5G illumination at a bias of 1.03 V (Figure 5c), significantly outperforming the control cell (Figure S19, Supporting Information). A certified efficiency of 26.29% is obtained using the strategy we proposed (Figure S20, Supporting Information), which is among the highest efficiencies reported for n‐i‐p single‐junction PSCs based on SnO_2_ (Table S6, Supporting Information). External quantum efficiency (EQE) spectrum shows that both control and target cells present high EQE values up to ≈94% in the visible light range (Figure 5d). The integrated *J*_sc_ of control and target cells is 25.34 and 25.53 mA cm^−2^ respectively, corresponding to over 95% of the *J*_sc_ values derived from *J–V* curves (Figure 5b; Figure S19, Supporting Information). Even though an increase in substrate transmittance was observed at the 300–500 nm range after the incorporation of AlCl_3_ (Figure S3, Supporting Information), no obvious increase in EQE was detected at this range. The discrepancy originates from the change of the reflection interface from the SnO_2_/air interface in the substrate to the SnO_2_/perovskite interface in the complete devices (Figure S21, Supporting Information). Apart from that, it is worth noting that the bandgap (1.53 eV) of perovskite is determined via EQE spectra and their differential spectra, rather than steady PL spectra (Figure S22, Supporting Information). It is because the PL peak position may be affected by the Stokes shift and/or test system, and an obvious difference between PL and EQE has been reported in the literature.^[^ ^2^, ^45^, ^46^, ^47^ ^]^ Large‐area PSCs with an active area of 1 cm^2^ were also fabricated, and the champion device exhibited PCEs of 25.44% (reverse scan) and 25.17% (steady‐state), outperforming the control device (Figure S23 and Table S7, Supporting Information). This demonstrates that our strategy is readily applicable to large‐area devices, highlighting its potential for scalable photovoltaic applications.

A series of characterizations were performed to investigate the mechanisms underlying the improvement in device performance. As shown in Figure 5e, the target cell showed a lower current under dark conditions as compared to the control cell. This indicates that the insert layer is beneficial to reduce the leakage current in the device. Besides, the target cell exhibited significantly higher recombination resistance (*R*_rec_) and lower charge transfer resistance (*R*_tr_) compared to the control cell, as shown in electrical impedance spectroscopy (EIS) results (Figure S24, Supporting Information). Specifically, the *R*_rec_ increased from 1.14 × 10^5^ to 1.15 × 10^6^ Ω cm^2^, and the *R*_tr_ decreased from 6495 to 4325 Ω cm^2^ after the incorporation of AlCl_3_. *R*_s_ remained almost identical in both cells (Table S8, Supporting Information). These results indicate that the inserted layer significantly suppresses the charge carrier recombination at the SnO_2_/perovskite interface and enhances carrier extraction and transport. This improvement is likely attributed to the passivation effect of Al_2_O_3_ on the perovskite layer. The ultra‐thin inserted layer also acts as a tunnelling layer for electrons, which may explain the unchanged *R*_s_ in both cells. The *J–V* curves of both control and target PSCs were measured under different light intensities (Figure S25a,b, Supporting Information). Photovoltaic parameters are summarized in Tables S9 and S10 (Supporting Information). The target cell exhibited much higher *V*_oc_ at various light intensities compared to the control cell, while the difference in *J*_sc_ between the cells was negligible (Figure S25c,d, Supporting Information). Furthermore, the deviation between the slope of *V*_oc_ and the value of (*k*_B_ *T*/*q*) decreased from 1.65 to 1.24 after the introduction of AlCl_3_ (Figure S25c, Supporting Information), indicating that trap‐assisted carrier recombination was suppressed at the interface.

The long‐term stability of the PSCs was measured to investigate how the AlCl_3_ layer impacts on the cell stability. As shown in Figure 5f, both control and target cells show good stability under dry air with relative humidity of ≈5% and at room temperature (≈25 °C). Specifically, the target cells maintained 93.88% of their initial efficiencies after 10 044 h of storage, while control cells retained 81.69% of their initial values. The photovoltaic parameters including *V*_oc_, *J*_sc_ and FF showed negligible change in target devices during the storage, but the efficiency (*V*_oc_ and FF) of control cells decreased from the initial 24.33% (1.161 V and 0.782) to 19.87% (1.106 V and 0.706) after 10044 h of storage (Figures S26 and S27, Supporting Information). Besides, the target cell showed significantly enhanced operational stability under light, as compared to the control cell. They retained 80% of their initial performance after 502 and 260 h of operation, respectively (Figure 5g). The improvement in device stability is likely to be due to the reduction of defects and hydroxyl groups at the SnO_2_/perovskite interface, which helps prevent the deprotonation of perovskites at the interface under external stimuli. We attempted to encapsulate our cells; however, the encapsulation process involves heating to 65 °C, which resulted in a decline in device performance (Figure S28 and Table S11, Supporting Information). While part of the decrease in *J*_sc_ can be attributed to the glass used for encapsulation, the reduction in FF occurred after encapsulation, indicating that some degradation of the PSCs was caused by the encapsulation process. It should be attributed to the instability of the doped spiro‐OMeTAD layer under elevated temperature, but it can be addressed by using alternative hole transport layers, such as copper phthalocyanine, poly(3‐hexylthiophen‐2,5‐diyl), and poly[bis(4‐phenyl)(2,4,6‐trimethylphenyl)amine].^[^ ^48^, ^49^, ^50^, ^51^ ^]^ While it is essential to introduce thermally stable hole transport layers into n‐i‐p cells, addressing this need extends beyond the focus of this work.

## 3 Conclusion

In summary, an effective and multifunctional strategy has been demonstrated to modify the SnO_2_/perovskite interface through the hydrolysis of AlCl_3_ and applying the resulting solution onto the SnO_2_ layer. This approach significantly enhances the coverage of rough substrates, thereby reducing the leakage current in PSCs. Furthermore, the hydrolysis reduces the surface potential of SnO_2_, leading to a better energy level alignment between SnO_2_ and the perovskite material. The hydrolysis, followed by neutralization reactions, effectively removes hydroxyl groups from the SnO_2_ surface, which mitigates the detrimental deprotonation of the perovskite induced by hydroxyls. Additionally, the formation of Al_2_O_3_ passivates defects at the SnO_2_/perovskite interface thereby significantly suppressing carrier recombination within the cells, while also acting as an electron tunnelling layer. This strategy substantially increases both the efficiency and stability of PSCs. Notably, this approach is applicable to a range of hydrolyzable alkali metal halides and organic compounds, leading to significant improvements in the quality of the interfaces between perovskite and various metal oxide charge transport layers, including SnO_2_, zinc oxide, and nickel oxide.

## 4 Experimental Section

### Materials

All materials were used directly after purchase. SnO_2_ precursor (15% in H_2_O colloidal dispersion, colorless and transparent) was ordered from Alfa Aesar. Glass/FTO substrates (type: A11‐HRT) were purchased from Suzhou ShangYang Solar Technology Co.,Ltd. HC(NH_2_)_2_I (FAI, 99.99%), CH_3_NH_3_Cl (MACl, 99.99%), CH_3_NH_3_Br (MABr, 99.99%), and phenethylammonium iodide (PEAI) were from Greatcell Solar. Lead iodide (PbI_2_, 99.99%) was from Tokyo Chemical Industry (TCI). *N,N*‐dimethylformamide (DMF, 99.8%), isopropanol (IPA, 99.5%), chlorobenzene (99.8%), dimethyl sulfoxide (DMSO, 99.9%), acetonitrile (99.8%), lithium bis‐(trifluoromethanesulfonyl)imide (Li‐TFSI, 99.95%), aluminum chloride (AlCl_3_, 99.999%), rubidium chloride (RbCl, 99.95%), [6,6]‐phenyl‐C61‐butyric acid methyl ester (PC_61_BM, >99%), and 4‐tert‐Butylpyridine (tBP, 98%) were purchased from Sigma‐Aldrich. 2,2′,7,7′‐Tetrakis‐(*N,N*‐di(4‐methoxyphenyl)amino)‐9,9′‐spirobifluorene (spiro‐OMeTAD, 99.8%) was from Lumtec.

### Device Fabrication

The Glass/FTO substrates were sequentially cleaned by sonication in detergent solution, deionized water, acetone, isopropanol, and ethanol for 30 min each. Substrates were then dried with nitrogen gas and treated with UV‐ozone for 30 min before use. The SnO_2_ colloidal dispersion was diluted by deionized water (SnO_2_: deionized water, volume ratio = 1: 5.309) and filtered using a 0.22 µm nylon filter. The diluted SnO_2_ precursor was spin‐coated onto the substrates at 3000 rpm for 30 s, followed by annealing at 150 °C for 30 min in ambient air. For target samples, AlCl_3_ (2 mg mL^−^^1^ in deionized water) was spin‐coated onto the SnO_2_ layer at 3000 rpm for 30 s, followed by annealing at 200 °C for 30 min in ambient air. All samples were subjected to UV‐ozone treatment for 30 min before the deposition of perovskite. It was noted that a large amount of white smoke and heat was generated when AlCl_3_ dissolves in water. It was not recommended to dissolve too much AlCl_3_ in water at one time. Two‐step spin‐coating in a glove box was utilized for the deposition of perovskite films. PbI_2_ (1.5 M) and RbCl (0.075 M) were dissolved in a mixed solvent (DMF: DMSO, volume ratio = 0.925: 0.075) and stirred vigorously at room temperature for 50 min. The precursor solution was spin‐coated at 1500 rpm for 30 s, followed by annealing at 70 °C for 1 min. The mixed solution for the second step was prepared with FAI: MABr: MACl (90 mg: 4 mg: 10 mg in 1 mL isopropanol). This solution was spin‐coated at 1800 rpm for 30 s, followed by annealing at 80 °C for 8 s in the glove box. Subsequently, all samples were transferred to ambient air (without controlling relative humidity) for annealing at 150 °C for 15 min. After cooling to room temperature, PEAI (4 mg mL^−^^1^ in isopropanol) was deposited onto the perovskite layer at 5000 rpm for 25 s with dynamic spin‐coating, during which the PEAI solution was dropped 5 s after the spin‐coating started. 72.3 mg of spiro‐OMeTAD was dissolved in 1 mL of chlorobenzene, in which 28.8 µL tBP and 17.5 µL Li‐TFSI solution (520 mg mL^−^^1^ in acetonitrile) were added. The mixed solution was filtered by a 0.22 µm polytetrafluoroethylene (PTFE) filter and then spin‐coated at 4000 rpm for 25 s on the perovskite film. A 100 nm Au electrode was prepared via thermal evaporation under a pressure of 8 × 10^−^^6^ mbar using a mask, resulting in an active area of 0.102 cm^2^. An anti‐reflection layer (MgF_2_, 100 nm) was thermally evaporated on the glass side of the PSCs, with the deposition rate of 0.3 nm s^−1^. For certification, a black shadow mask was used to define the effective working area of the PSCs as 0.0660 cm^2^. It should be noted that the shadow mask was not employed during the regular measurements. Applying a shadow mask results in an ≈25 mV reduction in *V*_oc_ and a ≈1.5% enhancement in FF compared to measurements without a shadow mask. (Figure S29 and Table S12, Supporting Information). A similar phenomenon had been reported in the literature.^[^ ^52^, ^53^, ^54^ ^]^

For the SCLC measurement, the samples have the structure of Glass/FTO/SnO_2_/perovskite/PC_61_BM/Au. PC_61_BM solution (20 mg mL^−^^1^ in chlorobenzene) was spin‐coated onto perovskite films at 3000 rpm for 30 s without further annealing. When PEAI was applied on the perovskite surface, the preparation process was identical to the PEAI deposition process described above.

For encapsulation, the cell was sandwiched between two pieces of super‐white (low‐iron) glass, and the perimeter was sealed with polyisobutylene (PIB). The assembly was then gradually heated to 65 °C under uniform pressure and cured in nitrogen for 3 days.

### Characterizations on Films

Scanning electron microscope (FEI Verios SEM system) was utilized to characterize the surface morphology of the substrates and perovskite films, and cross‐section images of PSCs. Atomic force microscopy, conductive AFM, and Kelvin probe force microscopy images were acquired using a Cypher ES scanning probe microscope with Pt‐coated Si tips. For current mapping, a 1 V DC bias was applied to the FTO electrode. An ultrahigh‐vacuum Kelvin probe system (KP Technology Φ4) was utilized to measure the work function of the substrates. The system's mapping mode was applied to assess the uniformity of the work function of the samples. Photoemission yield was measured under vacuum via the same Kelvin probe system to obtain the VBM of perovskite and spiro‐OMeTAD layers. The transmittance of the substrates was measured using a Lambda 1050 UV/Vis/NIR spectrophotometer (Perkin Elmer) in integrating sphere mode, with light incident on the glass side. Element distribution and XPS spectra of Glass/FTO/SnO_2_ were obtained with an ESCALAB250Xi using mono‐chromated Al K alpha radiation (1486.68 eV) under a pressure below 2 × 10^−^^9^ mbar. Depth profiling was performed by etching with a 1 keV Ar ion beam over a 2.5 × 2.5 mm area at 0.18 nm s^−1^. UPS spectra were acquired using an ESCALAB250Xi (Thermo Scientific, UK) with ultraviolet light (He I, 21.22 eV) under a pressure below 2 × 10^−9^ mbar. During the measurement, a bias voltage of −5 V was applied to the sample. To obtain the depth profile, an Ar ion beam (1 keV) was employed for etching. The etching area was 5 mm × 5 mm, with a reference etching rate of 0.04 nm s^−1^. The analysis of depth‐dependent UPS spectra should combine with the information provided by SEM cross‐section images, in which the thickness of perovskite films is ≈750 nm and the thickness of the area mixing with SnO_2_ and perovskite is ≈81 nm. Thus, the actual etching rate for perovskite and the mixing area is 0.20 and 0.05 nm s^−1^, respectively. Actual etching rate for SnO_2_ and underneath transparent conductive oxide is 0.04 nm s^−1^, which was consistent with the previous results.^[^ ^18^ ^]^

The GIWAXS experiments were completed at beamline BL03HB of the Shanghai Synchrotron Radiation Facility using an X‐ray wavelength of 0.124 nm with a spot diameter of ≈3 µm. The incident angle was 0.3° with an exposure time for each collection of GIWAXS data set to be 5 s. Grazing incidence X‐ray diffraction was performed using a D2 Phaser X‐ray Diffractometer with a step size of 0.02° and an integration time of 0.4 s per step, employing Cu Kα radiation as the X‐ray source. The measurements were taken from the front side of the samples, using silicon as the substrate, with an incident angle of 0.6°. The cross‐section used for TEM images was prepared by a Helios Nanolab 600 focused ion beam (FIB) system. An amorphous carbon layer and a Pt layer were deposited at the surface of the Glass/FTO/SnO_2_/AlCl_3_ sample to hold the cross‐section during FIB etching process. The TEM measurements were performed in a JEOL 2100F. The calculation of lattice spacings involves the use of fast Fourier transform and inverse fast Fourier transform through the ImageJ software. Time‐resolved photoluminescence (TRPL) was measured using a Horiba iHR 320 mm spectrophotometer with a compact single‐photon silicon detector (PPD‐900, detection range between 350 and 920 nm). A 485 nm picosecond pulse laser (Horiba DeltaDiode) with a pulse width of 80 ps was used as the excitation source. Both TRPL and steady photoluminescence measurements were conducted on samples with the structure Glass/FTO/SnO_2_/perovskite, with the pulse laser incident from the glass side. A bi‐exponential decay function was employed to fit the TRPL decay, and the carrier lifetime was extracted accordingly.

### Characterizations on Solar Cells and Devices

ToF‐SIMS was conducted with Cs^+^ ions (1 keV, 72.9 nA) for erosion and Bi^3+^ ions (30 keV, 0.962 pA) for analysis over a 100 × 100 µm area with negative polarity. PL images of PSCs were taken using a custom PL imaging system under 0.5 sun illumination (≈50 mW cm^−^^2^) and open–circuit conditions. A Peltier‐cooled (−70 °C) Si CCD camera (Princeton Instruments Pixis 1024) with a long‐pass filter (750 nm) automatically recorded images every 10 s from PSCs with the exposure time of 20 ms. *J–V* curves of PSCs were measured from +1.2 to −0.05 V at 0.15 V s^−^^1^ and 10 mV interval using a potentiostat source (AutolabPGSTAT302N) and solar simulator (#WAVELABS SINUS‐220), with light intensity calibrated to 100 mW cm^−^^2^ using a Fraunhofer CalLab reference cell. PSCs underwent short light soaking (20–40 s) before testing at ≈25 °C under nitrogen flow. Steady‐state efficiency was measured under one sun illumination by applying a bias voltage near the maximum power point after *V*_oc_ stabilization, and the current was monitored accordingly. EQE spectra and reflectance spectra were measured with an Enlitech (QE‐R) system. An anti‐reflection layer (MgF_2_, 100 nm) was thermally evaporated on the glass side for EQE measurement. Dark current–voltage (*I*–*V*) curves of PSCs were measured using a KEYSIGHT B2902A Precision Source/Measure Unit from −0.4 to +1.2 V, with a 5‐mV interval and a 0.1 s measurement delay, under dark conditions and ambient air. The SCLC of electron‐only samples was measured using the same KEYSIGHT unit from 0 to 1.5 V, with a 1 mV interval and a 0.1 s measurement delay, under dark conditions and ambient air. Nyquist plots were obtained by Metrohm Autolab (PGSTAT302N) under dark conditions without bias voltage, covering a frequency range from 0.1 to 1 × 10^5^ Hz; data was fitted and analyzed using ZSimpWin. Operational stability tests were conducted on unencapsulated PSCs in an N_2_‐filled glove box at ≈25 °C using a HYPERION solar simulator (HYPIV20 system), while applying a bias voltage near the initial maximum power point and tracking current density with a UV filter. Outliers in the operational stability data were removed using a Rolling Median Filter, followed by averaging every 20 data points. The dry box for PSCs storage maintained a relative humidity of ≈3%, rising above 30% when opened, averaging ≈5%.

### Statistical Analysis

Statistical analyses were conducted using Origin 2021. *V*_oc_ and FF were rounded to three decimal places, while *J*_sc_ and PCE were rounded to two. Photovoltaic parameters for control and target PSCs were plotted for over 20 devices of each type (Figure 5a). Data from AFM, c‐AFM, KPFM, XPS, ToF‐SIMS, SEM, PL images, steady PL, *J–V*, EQE, and steady‐state efficiency were shown as original, non‐normalized data. Fittings were applied to *V*_oc_ and *J*_sc_ versus light intensity, TRPL spectra, photoemission yield, and the SCLC.

## Conflict of Interest

The authors declare no conflict of interest.

## Supporting information



Supporting Information

Supplemental DataFile 1

Supplemental DataFile 2

## Data Availability

The data that support the findings of this study are available from the corresponding author upon reasonable request.
