# Supplementary material for: Multifunctional SnO2/Perovskite Interface Engineering for Efficient Perovskite Solar Cells
Source: Adv Sci (Weinh). 2025 Sep 26;12(46):e14595. doi: 10.1002/advs.202514595 (PMC12697791; doi:10.1002/advs.202514595)
Supplement: Supplementary file 1 — Supporting Information [file ADVS-12-e14595-s002.pdf]

Supporting Information

Multifunctional SnO_2/perovskite interface engineering for efficient perovskite solar cells

Keqing Huang, Wei Wang, Anh Dinh Bui, Wenzhong Ji, Felipe Kremer, Zhongshu Yang, Gabriel Bartholazzi, Yang Yu, Olivier Lee Cheong Lem, Bingchen He, Zhenhuang Su, Viqar Ahmad, Lichun Chang, Dang-Thuan Nguyen, Yun Liu, Xingyu Gao, Junliang Yang, Kylie R. Catchpole, Heping Shen,* Klaus J. Weber,* The Duong*

K. Huang, W. Wang, A. D. Bui, Z. Yang, G. Bartholazzi, V. Ahmad, L. Chang, D.-T. Nguyen, K. R. Catchpole, H. Shen, K. J. Weber, T. Duong. School of Engineering, The Australian National University, Canberra, Australian Capital Territory 2601, Australia.
E-mail: heping.shen@anu.edu.au; klaus.weber@anu.edu.au; the.duong@anu.edu.au
W. Ji, Y. Liu. Research School of Chemistry, The Australian National University, Canberra, Australian Capital Territory 2601, Australia.
F. Kremer. Centre for Advanced Microscopy, The Australian National University, Canberra, Australian Capital Territory 2600, Australia.
Y. Yu. Research School of Physics, The Australian National University, Canberra, Australian Capital Territory 2600, Australia.
O. L. C. Lem. Australian National Fabrication Facility, Research School of Physics, The Australian National University, Canberra, Australian Capital Territory 2600, Australia.
B. He, Z. Su, X. Gao. Shanghai Synchrotron Radiation Facility (SSRF), Shanghai Advanced Research Institute, Chinese Academy of Sciences, Shanghai 201204, P. R. China.
J. Yang. Hunan Key Laboratory for Super-microstructure and Ultrafast Process, School of Physics, Central South University, Changsha 410083, P. R. China.

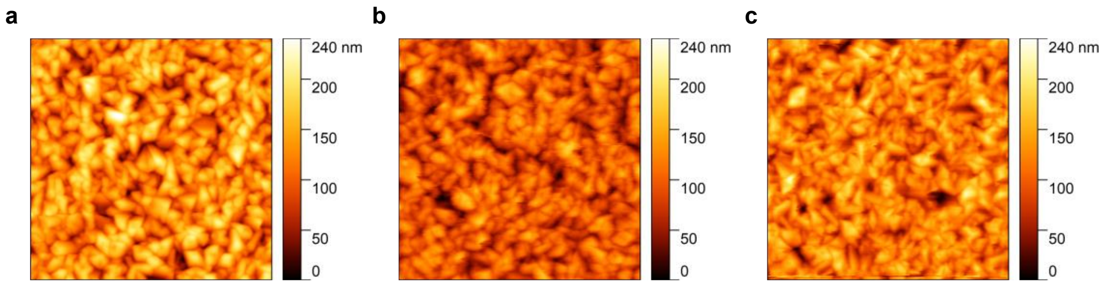

Figure S1. AFM height images (5 μm × 5 μm) of (a) FTO, (b) FTO/SnO_2, and (c) FTO/SnO_2/$AlCl_3$. It is noted that the root-mean-square roughness of the FTO, FTO/SnO_2, and FTO/SnO_2/$AlCl_3$ samples is 36.6, 27.4, and 29.2 nm, respectively.

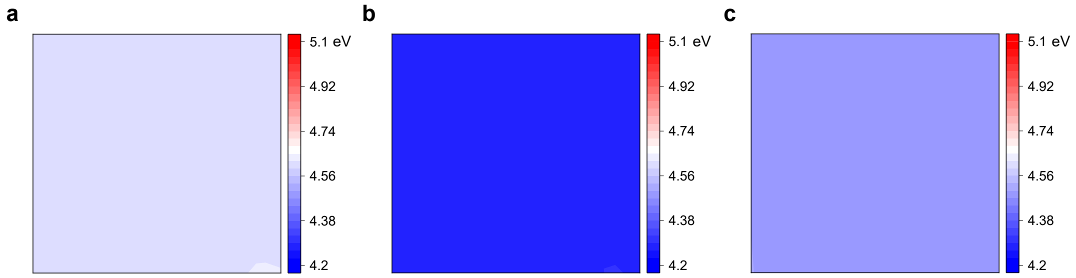

Figure S2. Work function maps (1 mm × 1 mm) of (a) FTO, (b) FTO/SnO_2, and (c) FTO/SnO_2/$AlCl_3$. It is noted that these images are derived from Kelvin Probe.

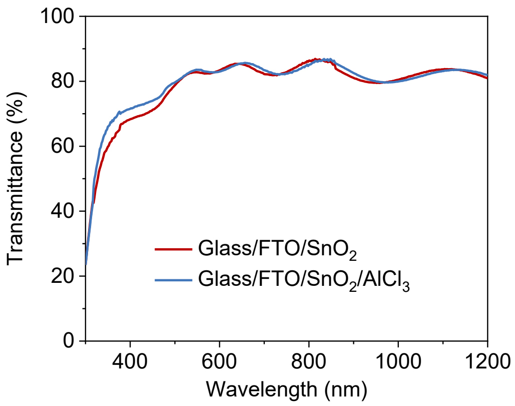

Figure S3. Transmittance spectra of the substrates with and without $AlCl_3$. It is noted that no antireflection layer (magnesium fluoride) was applied in these transmittance spectra.

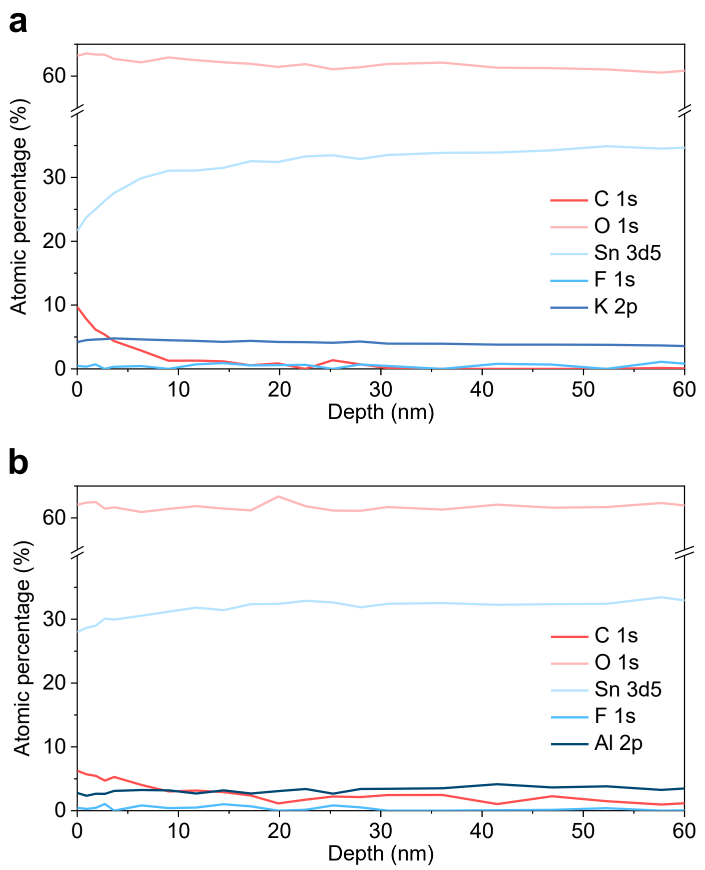

Figure S4. Depth dependence of element distribution of (a) Glass/FTO/SnO_2 and (b) Glass/FTO/SnO_2/$AlCl_3$ samples.

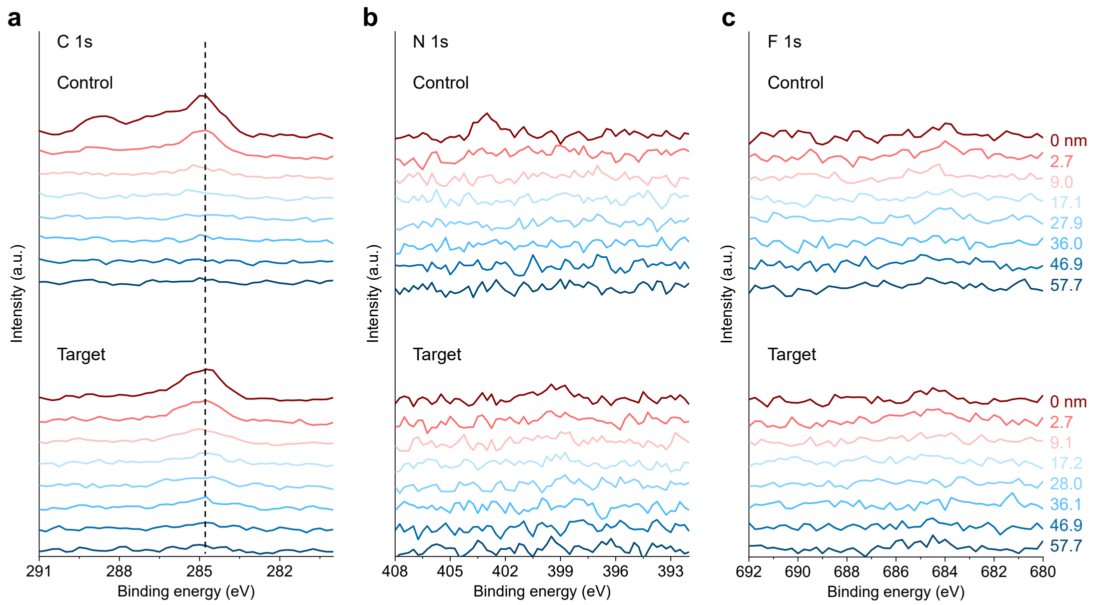

Figure S5. Depth-dependent XPS spectra of (a) C 1s, (b) N 1s, and (c) F 1s of control and target Glass/FTO/SnO_2 samples.

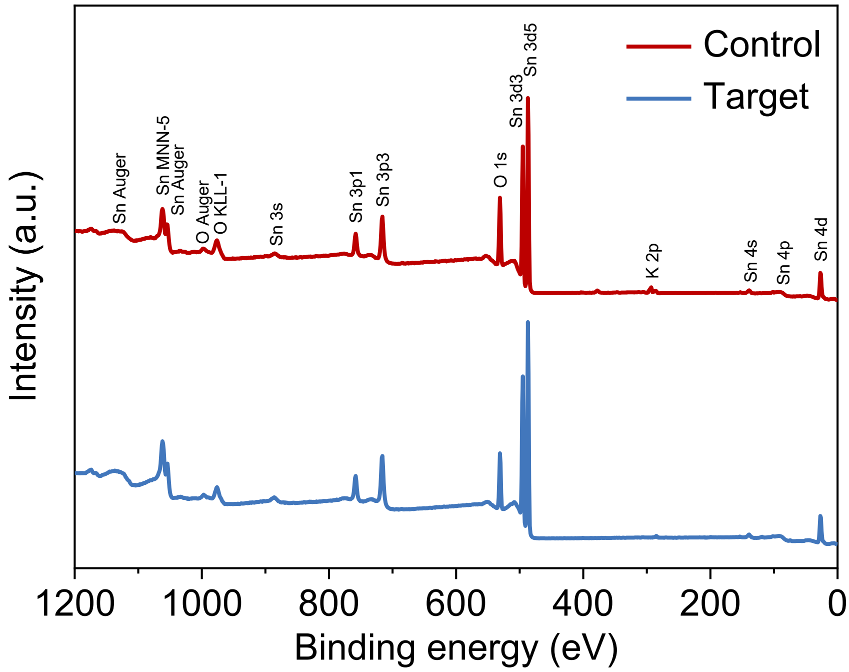

Figure S6. Full XPS spectra of control and target Glass/FTO/SnO_2 samples.

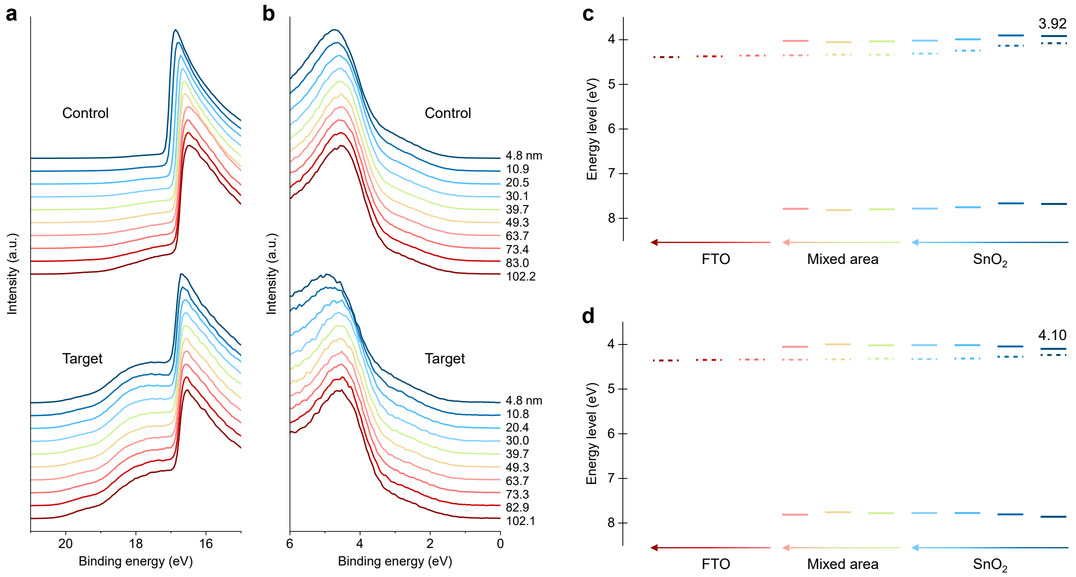

Figure S7. Depth-dependent UPS spectra of control (Glass/FTO/SnO_2) and target (Glass/FTO/SnO_2/$AlCl_3$) samples, showing (a) secondary electron cut-off and (b) valance band regions; energy level alignment of (c) control and (d) target samples.

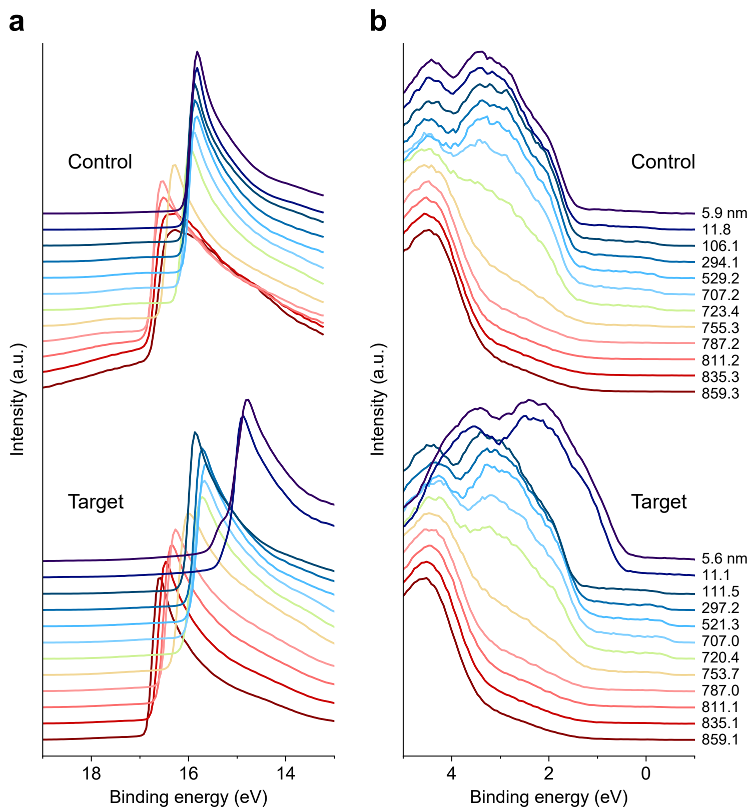

Figure S8. Depth-dependent UPS spectra of control (Glass/FTO/SnO_2/perovskite) and target (Glass/FTO/SnO_2/$AlCl_3$/perovskite) samples, showing (a) secondary electron cut-off and (b) valance band regions.

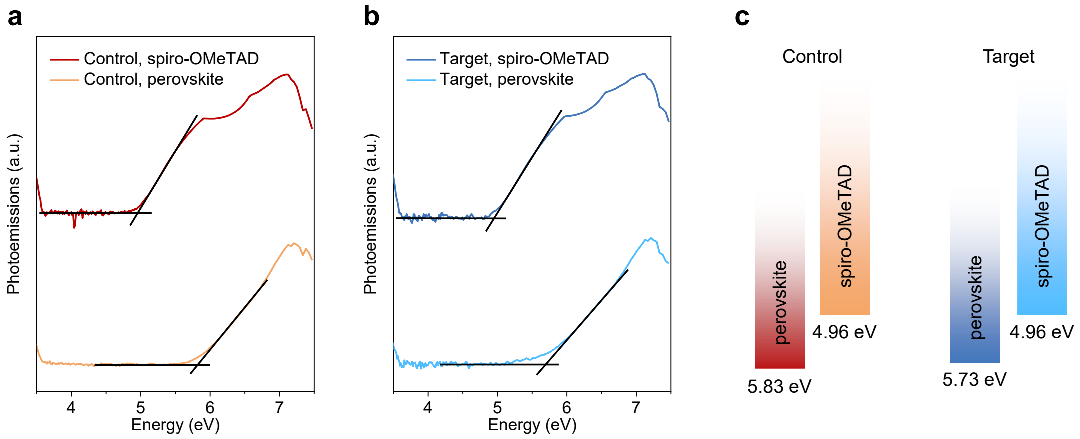

Figure S9. Photoemission yield showing extraction of VBM of perovskite and spiro-OMeTAD layers, (a) without $AlCl_3$ and (b) with $AlCl_3$ at the SnO_2/perovskite interface; (c) energy level alignment between perovskite and spiro-OMeTAD.

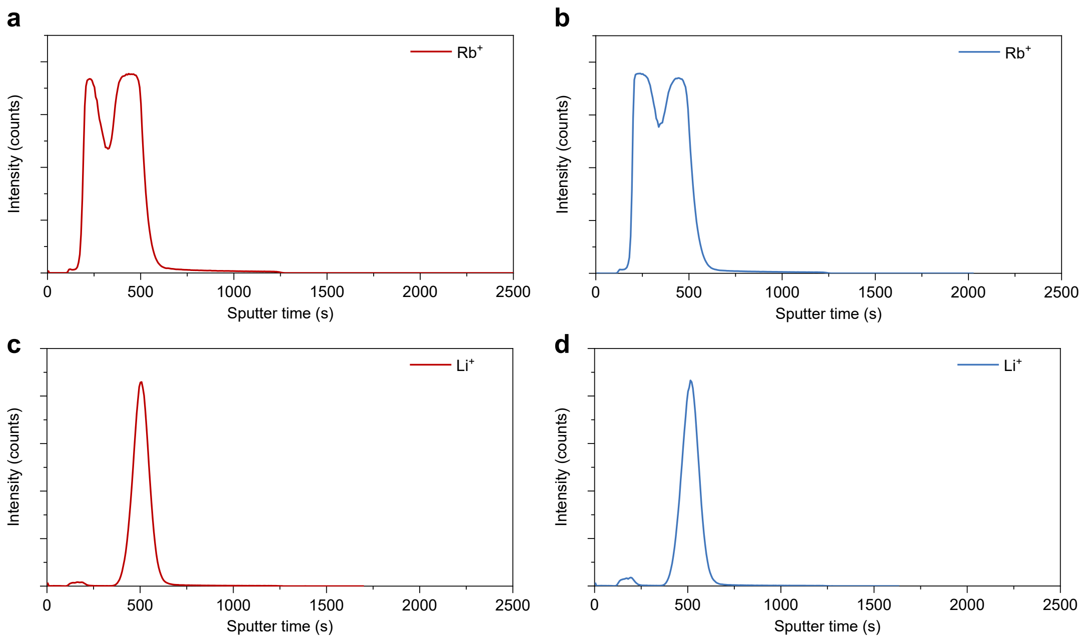

Figure S10. Rb element distribution of (a) control and (b) target PSCs; Li element distribution of (c) control and (d) target PSCs.

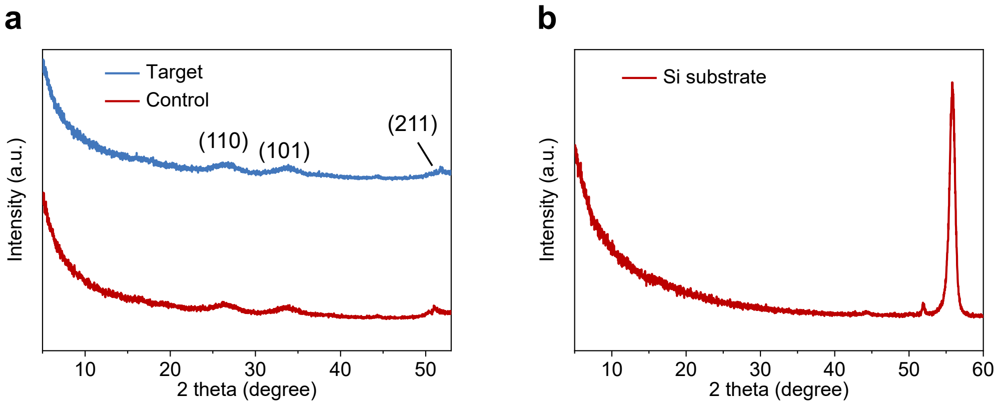

Figure S11. (a) GIXRD patterns of control (Si/SnO_2) and target (Si/SnO_2/$AlCl_3$) samples; (b) GIXRD pattern of bare silicon substrate.

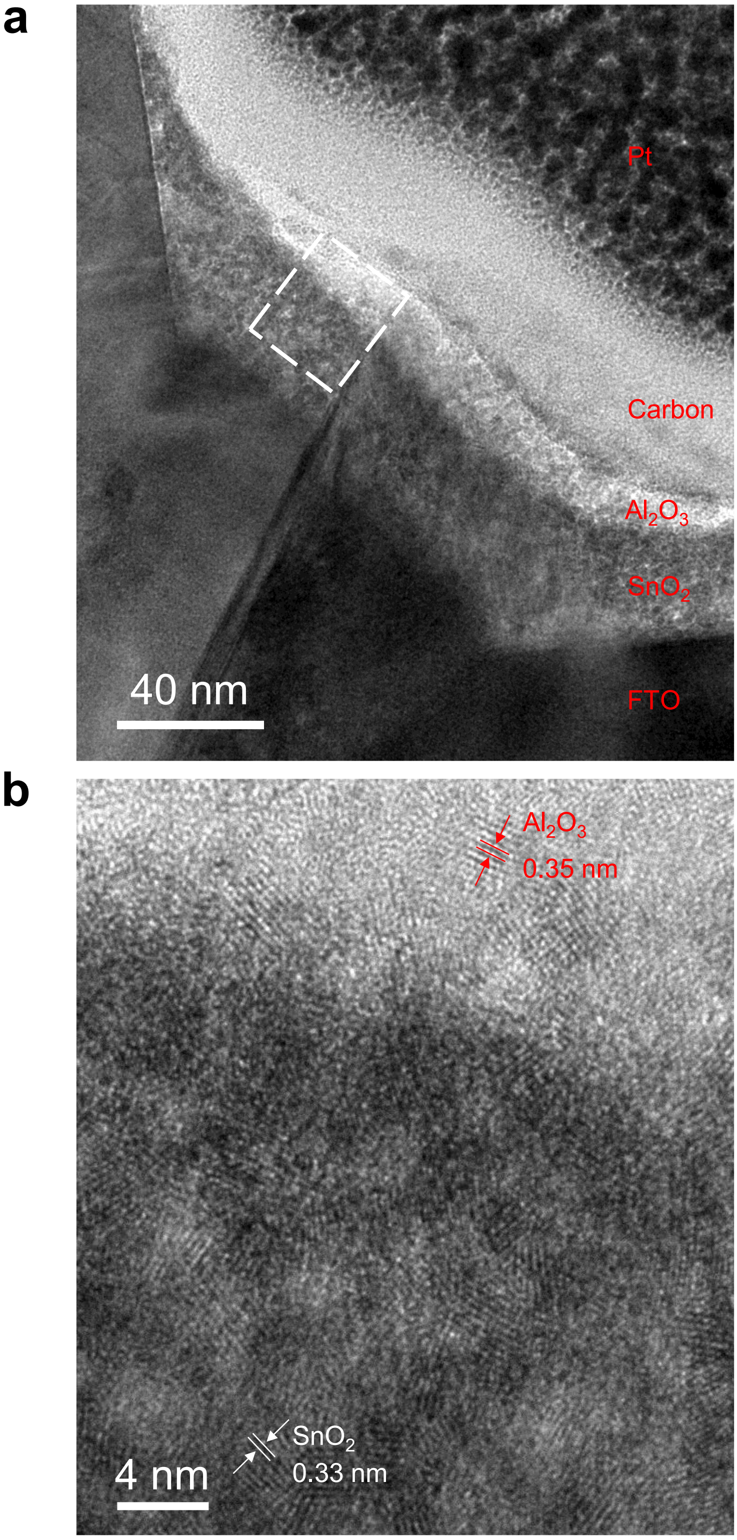

Figure S12. (a) and (b) TEM cross-section images of Glass/FTO/SnO_2/$AlCl_3$. Image b is derived from the dashed line range of image a. An amorphous carbon layer and a Pt layer were deposited at the surface of the sample, which were used to hold the cross section during FIB etching process.

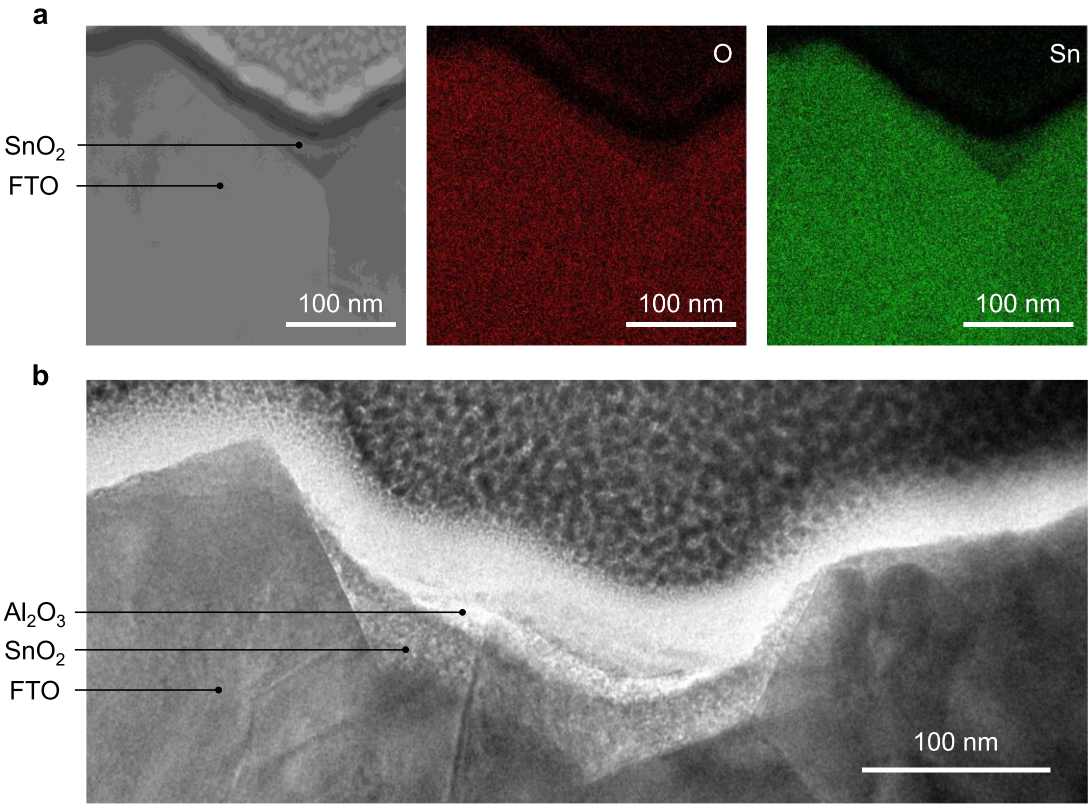

Figure S13. (a) STEM DF cross-section image and energy-dispersive X-ray spectroscopy mapping of Glass/FTO/SnO_2 sample; (b) TEM cross-section image of Glass/FTO/SnO_2/$AlCl_3$ sample.

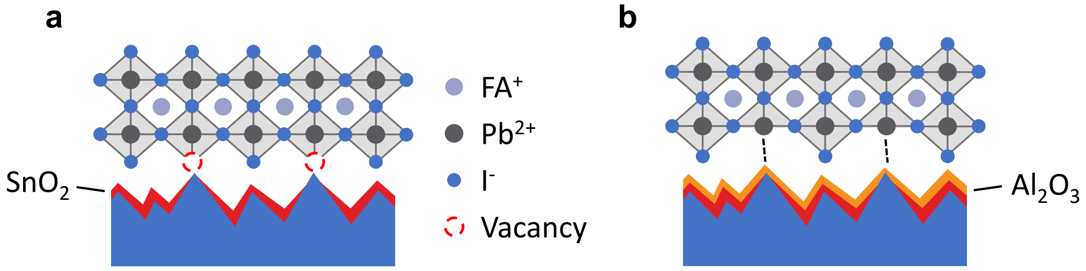

Figure S14. Schematic of (a) control and (b) target SnO_2/perovskite interface.

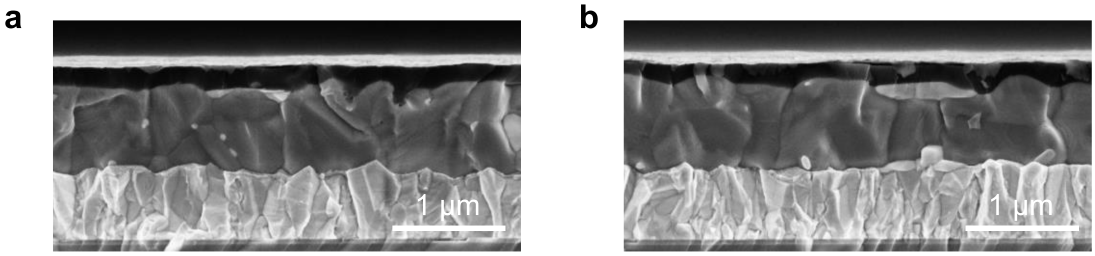

Figure S15. SEM cross-section images of (a) control and (b) target PSCs.

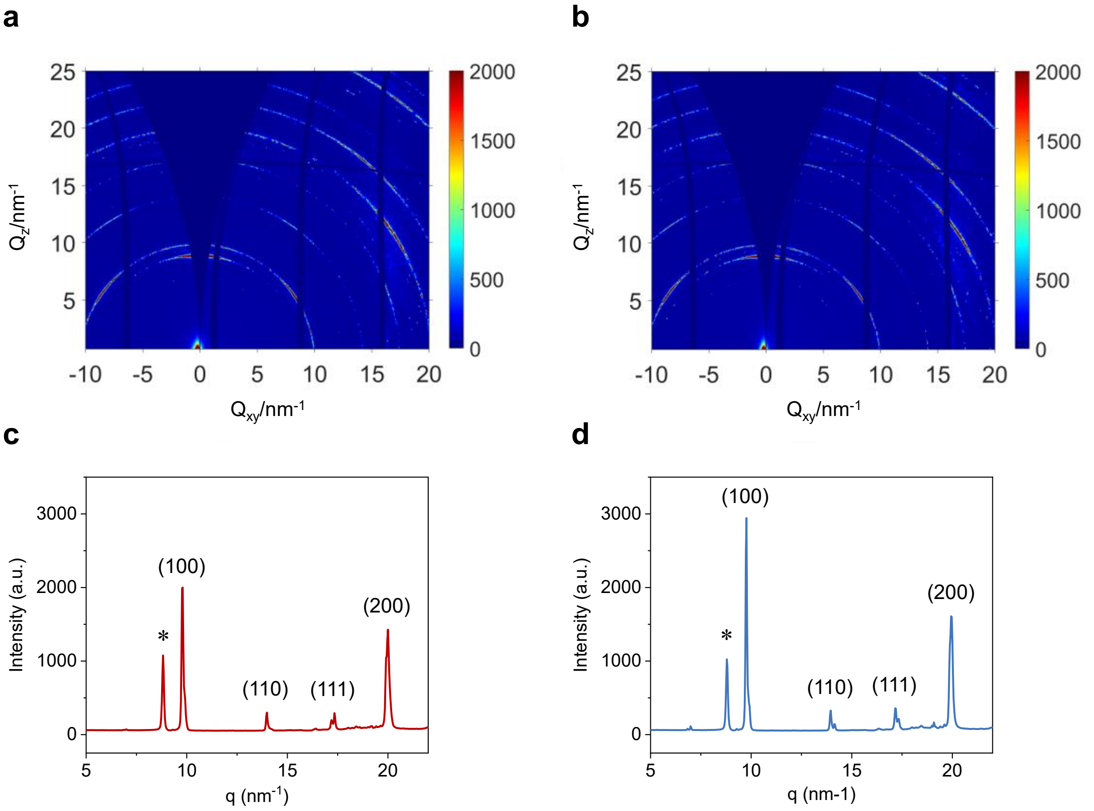

Figure S16. Two-dimensional GIWAXS patterns of (a) control and (b) target perovskite films; one-dimensional diffraction patterns of (c) control and (d) target perovskite films (* corresponds to the diffraction pattern of PbI_2).

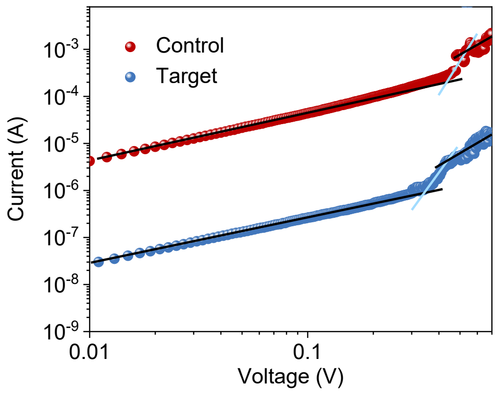

Figure S17. Current-voltage curves of the electron-only devices measured in the dark, with the PEAI passivation layer for the perovskite surface.

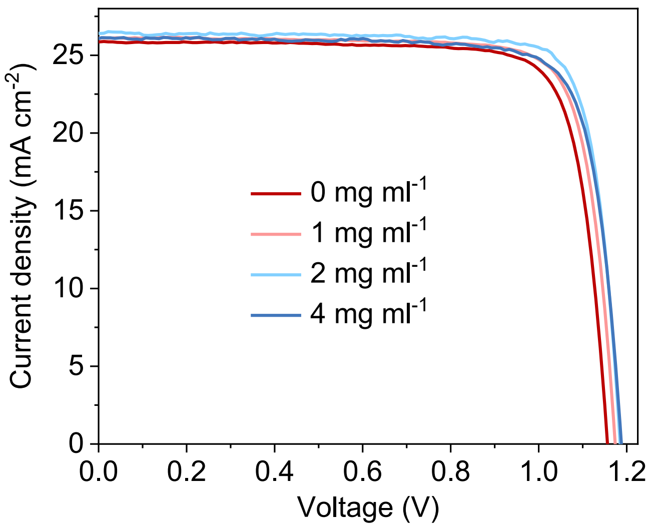

Figure S18. *J–V* curves of PSCs with different concentrations of $AlCl_3$.

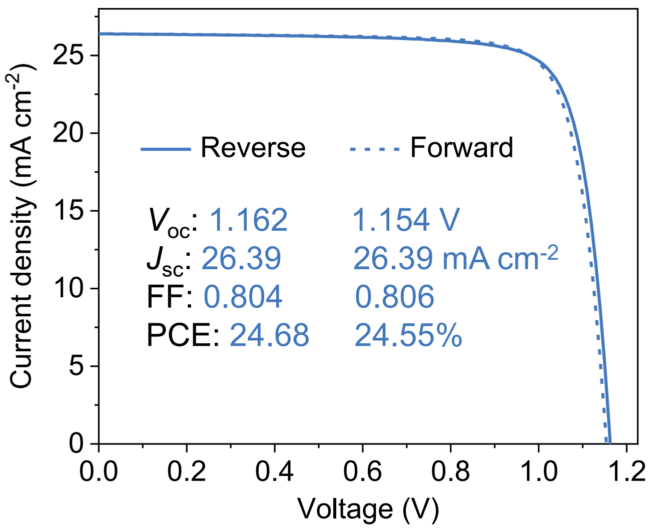

Figure S19. *J–V* curves of the champion control PSC under AM 1.5G illumination.

Test Report: I-V

PV PERFORMANCE LABORATORY
www.csiro.au

CSIRO

Report Date: 11 October 2024

Australian National University

NATA Accredited Laboratory
Number: 165
Corporate Site 22466
Accredited for compliance with
ISO/IEC 17025 - Testing

NATA

TEST RESULTS : A0030-ANU-D19_001

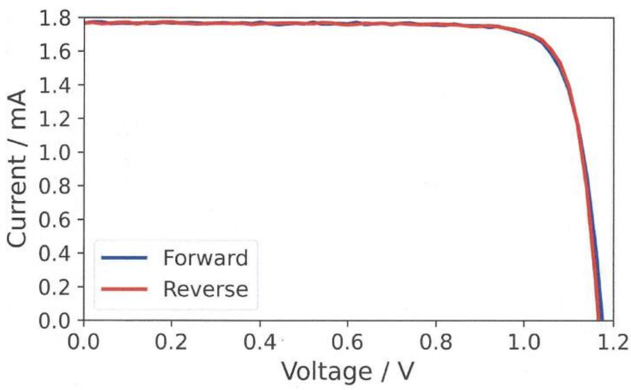

Forward	Reverse
**Open-circuit voltage:** 1.174 ± 0.011 V	**Open-circuit voltage:** 1.166 ± 0.011 V
**Short-circuit current:** 1.766 ± 0.059 mA	**Short-circuit current:** 1.765 ± 0.059 mA
**Maximum power:** 1.723 ± 0.060 mW	**Maximum power:** 1.735 ± 0.060 mW
**Fill factor:** 83.1 ± 1.2 %	**Fill factor:** 84.3 ± 1.2 %
**Efficiency:** 26.11 ± 0.92 %	**Efficiency:** 26.29 ± 0.92 %

Results are expressed at Standard Test Conditions (cell temperature 25 °C and spectral irradiance AM1.5G per IEC 60904-3, 1000 W/m^2). Uncertainty values shown are expanded absolute uncertainties expressed with a coverage factor of k=2. The illuminated area was 0.0660 ± 0.0003 cm^2, as defined by an aperture mask mounted on the cell by the customer.

Customer address:
32 North Road
Acton Canberra
2601 Australia

Test location:
PV Performance Lab
CSIRO Energy Centre
10 Murray Dwyer Cct
Mayfield West NSW
2304 Australia

**Authorised by:**	**Verified by:**
Benjamin Duck, Measurement Scientist	Chris Fell, PVPL Manager
Date: 02/12/2024	Date: 2/12/2024
Signature: [signature]	Signature: [signature]

Figure S20. Certified results from an accredited photovoltaic certification laboratory (Commonwealth Scientific and Industrial Research Organization (CSIRO), Australia), showing the efficiency of 26.29%. The active area of the cell was 0.102 cm^2, and a black shadow mask with an aperture area of 0.0660 cm^2 was used.

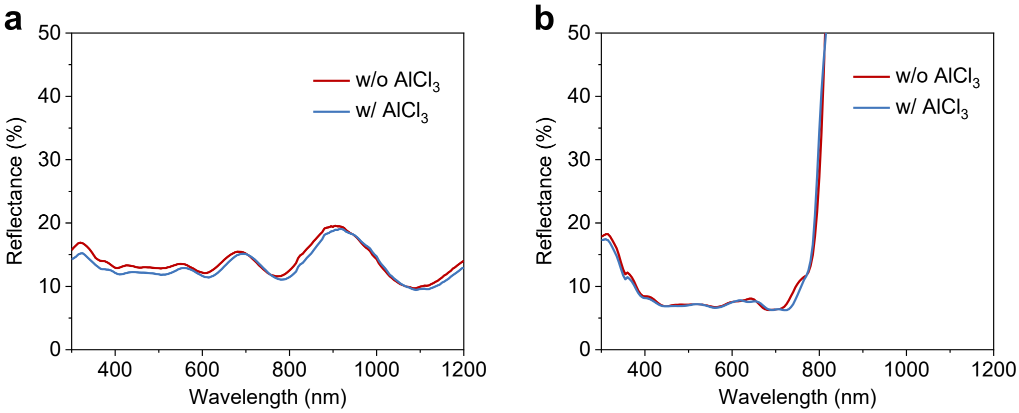

Figure S21. Reflectance spectra of (a) Glass/FTO/SnO_2 and (b) cells with and without $AlCl_3$. It should be noted that light was incident from the glass side, and no antireflection layer (magnesium fluoride) was applied in these reflectance spectra.

For the Glass/FTO/SnO_2 substrate, the rough FTO surface induces pronounced diffuse reflection at the SnO_2/air interface, resulting in low transmittance. After the incorporation of $AlCl_3$, a slight change in substrate reflectance was observed, particularly a decrease in the 300–500 nm range, which leads to a corresponding increase in transmittance (Figure S3, Supporting Information). In the complete devices, however, light reflection occurs at the SnO_2/perovskite interface. Owing to the high absorption coefficient of perovskite and its large refractive index contrast with air, the overall reflectance is significantly suppressed in the visible region (Figure S21, Supporting Information). Notably, no measurable difference in reflectance was observed in the 300–500 nm range for the final devices (Figure S21b, Supporting Information), which explains the negligible variation in EQE spectra.

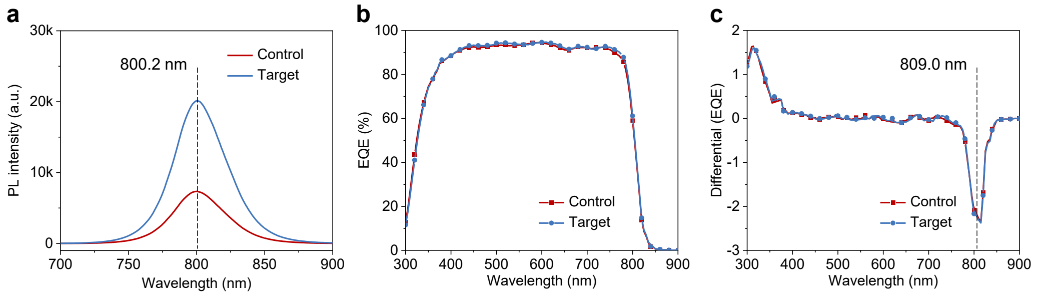

Figure S22. (a) Steady PL spectra of control and target perovskite films; (b) EQE spectra of control and target PSCs; (c) differential spectra of EQE.

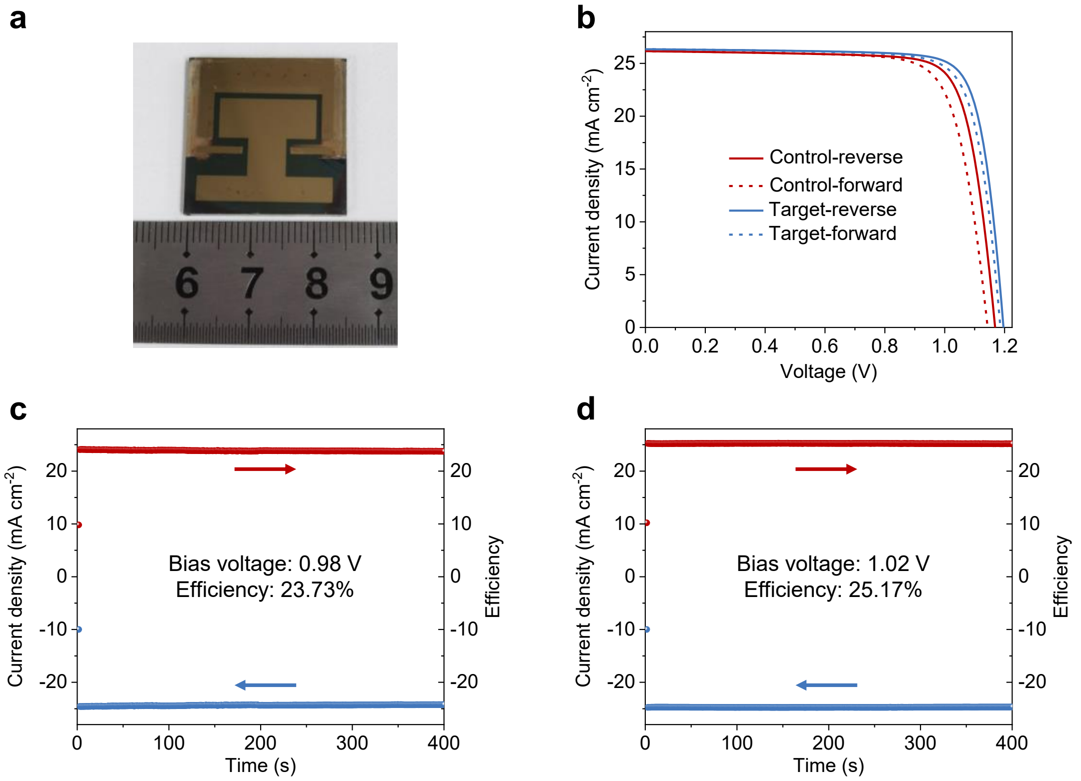

Figure S23. (a) Photograph of the large-area device (2.5 × 2.5 cm) with an active area of 1 cm^2; (b) *J–V* curves of the large-area control and target PSCs; steady-state efficiency of the large-area (c) control and (d) target cells.

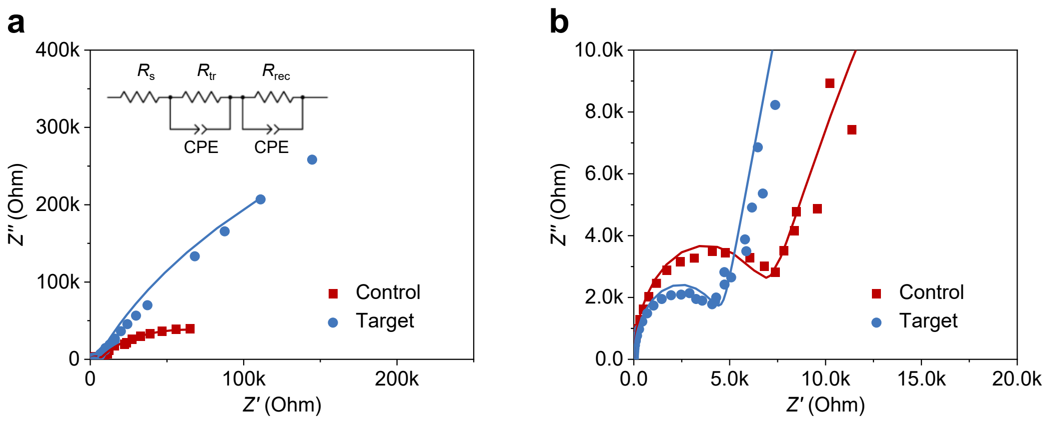

Figure S24. Nyquist plots in (a) low-frequency and (b) high-frequency region of control and target PSCs. Inset in image a is the equivalent circuit used to fit the data.

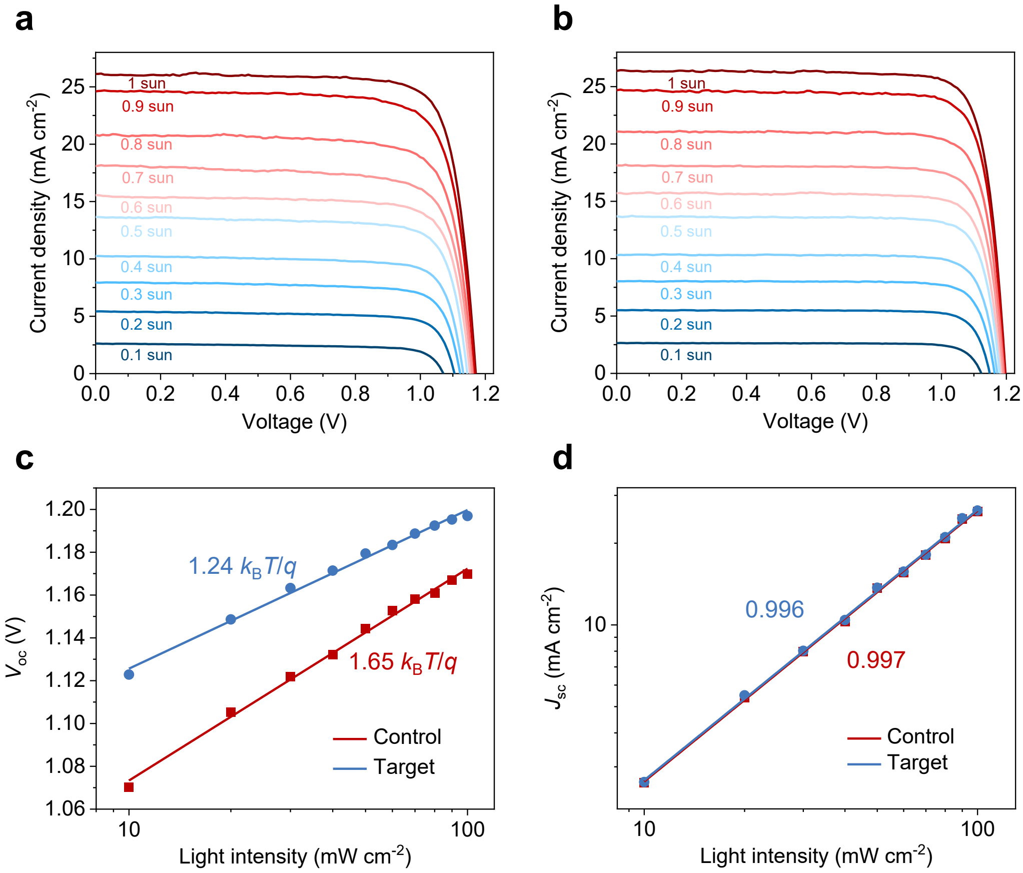

Figure S25. *J*–*V* curves of the (a) control and (b) target PSCs under different light intensities ranging from 0.1 sun to 1 sun; (c) relationship between V_{oc} and light intensity for the control and target PSCs; (d) relationship between J_{sc} and light intensity for the control and target PSCs.

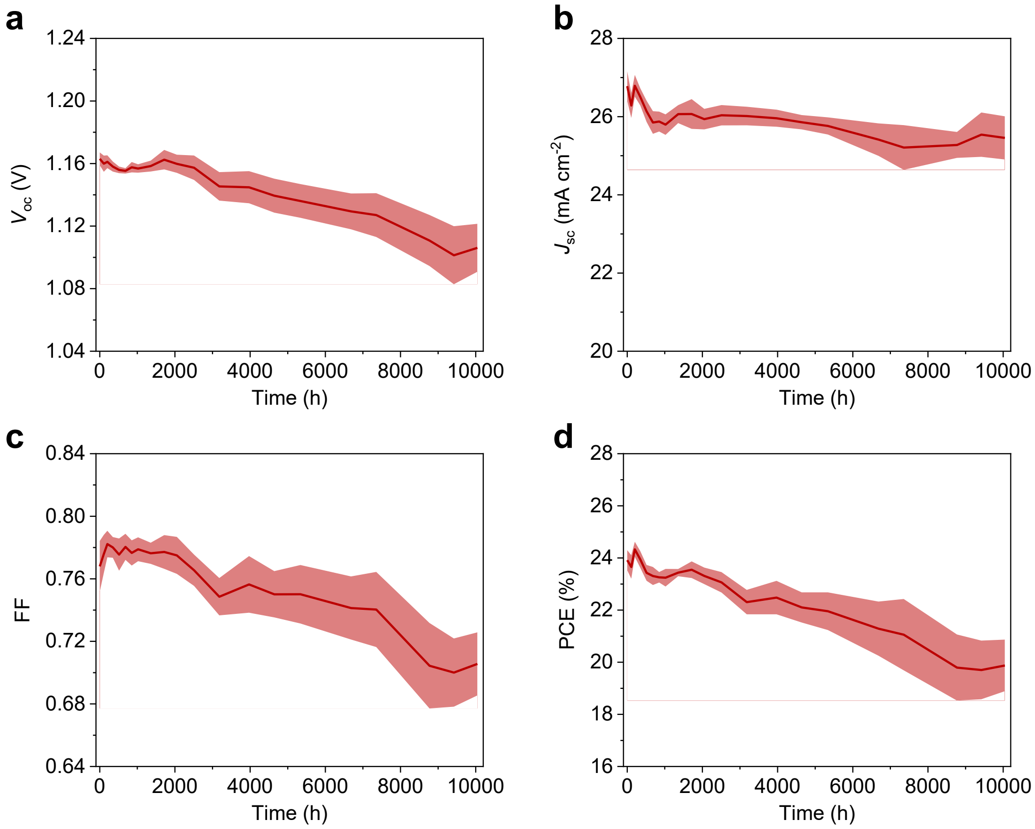

Figure S26. (a) V_{oc}, (b) J_{sc}, (c) FF, and (d) PCE of control PSCs stored in dry air (relative humidity of ~5% and ~25 °C). The results are based on 6 cells.

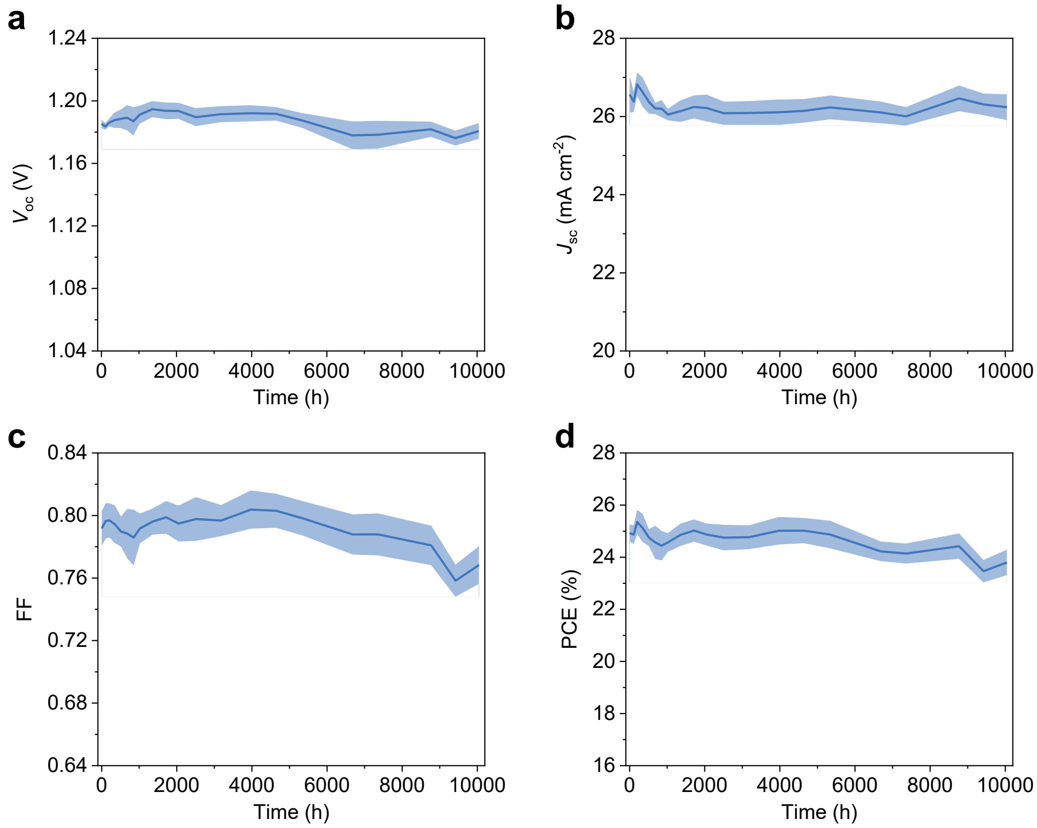

Figure S27. (a) V_{oc}, (b) J_{sc}, (c) FF, and (d) PCE of target PSCs stored in dry air (relative humidity of ~5% and ~25 °C). The results are based on 6 cells.

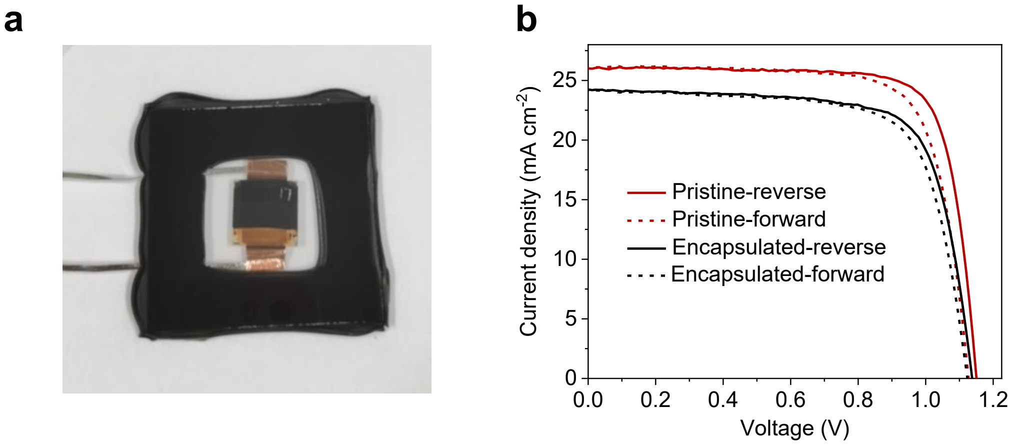

Figure S28. (a) Photograph of the encapsulated control cell; (b) *J–V* curves of the control cell before and after encapsulation.

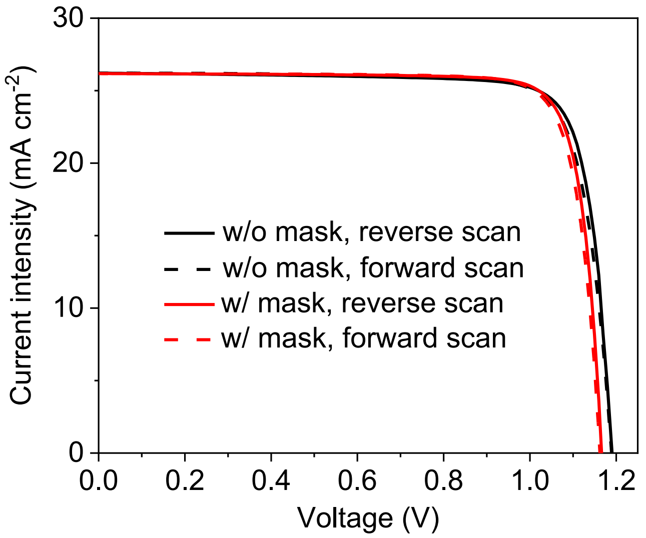

Figure S29. *J*–*V* curves of the target cell with and without a shadow mask during measurement.

Table S1. Data from depth-dependent UPS results of Glass/FTO/SnO_2 (control) and Glass/FTO/SnO_2/$AlCl_3$ (target) samples.

Depth (nm)	Cut-off	E_F-E_V	E_F	VBM	CB
Control-4.8	17.14	3.60	4.08	7.68	3.92
10.9	17.09	3.53	4.13	7.67	3.91
20.5	16.97	3.51	4.25	7.75	3.99
30.1	16.91	3.47	4.31	7.78	4.02
39.7	16.88	3.46	4.34	7.80	4.04
49.3	16.89	3.48	4.33	7.82	4.06
63.7	16.87	3.44	4.35	7.79	4.03
73.4	16.87	--	4.35	--	--
83.0	16.85	--	4.37	--	--
102.2	16.83	--	4.39	--	--
Target-4.8	16.98	3.62	4.24	7.86	4.10
10.8	16.94	3.53	4.28	7.81	4.05
20.4	16.90	3.46	4.32	7.78	4.02
30.0	16.89	3.45	4.33	7.78	4.02
39.7	16.90	3.46	4.32	7.78	4.02
49.3	16.89	3.42	4.33	7.76	4.00
63.7	16.88	3.47	4.34	7.81	4.05
73.3	16.88	--	4.34	--	--
82.9	16.87	--	4.35	--	--
102.1	16.86	--	4.36	--	--

Table S2. Data from depth-dependent UPS results of Glass/FTO/SnO_2/perovskite (control) and Glass/FTO/SnO_2/$AlCl_3$/perovskite (target) samples.

Depth (nm)	Cut-off	E_F-E_V	E_F	VBM	CB
Control-5.9	16.09	1.47	5.13	6.60	5.07
11.8	16.09	1.46	5.13	6.59	5.06
106.1	16.13	1.42	5.09	6.52	4.98
294.1	16.14	1.43	5.08	6.52	4.98
529.2	16.10	1.45	5.12	6.57	5.04
707.2	16.20	1.46	5.02	6.48	4.94
723.4	16.26	1.45	4.96	6.41	4.87
755.3	16.58	3.45	4.64	8.09	4.33
787.2	16.83	3.44	4.39	7.83	4.07
811.2	16.86	3.42	4.36	7.79	4.03
835.3	16.82	3.40	4.40	7.80	4.04
859.3	16.78	--	4.44	--	--
Target-5.6	15.30	0.42	5.92	6.34	4.80
11.1	15.25	0.48	5.97	6.45	4.92
111.5	16.12	1.40	5.10	6.50	4.97
297.2	15.99	1.31	5.23	6.54	5.00
521.3	15.98	1.24	5.24	6.48	4.95
707.0	16.05	1.19	5.17	6.36	4.82
720.4	16.12	1.14	5.10	6.24	4.71
753.7	16.35	3.49	4.87	8.36	4.60
787.0	16.58	3.39	4.64	8.03	4.27
811.1	16.64	3.38	4.58	7.96	4.20
835.1	16.75	3.43	4.47	7.90	4.14
859.1	16.85	--	4.37	--	--

Table S3. Fitted parameters of TRPL spectra for control and target samples. TRPL spectra were fitted by the equation: $\mathrm{y} = A_1 * \exp\left(-\frac{\mathrm{x}}{\tau_1}\right) + A_2 * \exp\left(-\frac{\mathrm{x}}{\tau_2}\right) + \mathrm{y}_0$. Average carrier lifetime (τ_{ave}) was calculated by the equation: $\tau_{\mathrm{ave}} = \frac{A_1\tau_1^2 + A_2\tau_2^2}{A_1\tau_1 + A_2\tau_2}$.

Sample	A_1	τ_1 (ns)	A_2	τ_2 (ns)	τ_{ave} (ns)
Control	0.38	61.45	0.58	299.05	271.05
Target	0.34	118.12	0.62	442.20	400.52

Table S4. Performance data for PSCs with different concentrations of $AlCl_3$.

Concentration (mg mL^{-1})	V_{oc} (V)	J_{sc} (mA cm^{-2})	FF	PCE (%)
0	1.156	25.88	0.805	24.09
1	1.174	26.12	0.809	24.82
2	1.185	26.50	0.824	25.87
4	1.188	26.05	0.805	24.92

Table S5. R_{sh} and R_s of PSCs with varying $AlCl_3$ concentrations at the SnO_2/perovskite interface. These values are extracted from reverse-scan *J–V* curves, with each condition based on five *J–V* curves.

Concentration (mg mL^{-1})	0	1	2	4
R_{sh} (Ω cm^2)	2574.03	3333.20	4364.81	2193.10
R_s (Ω cm^2)	0.45	0.34	0.34	0.30

Table S6. A summary of recent progress of n-i-p single-junction PSCs.

Cell configuration	Key point	Perovskite coating method	Active area (cm^2)	PCE (%)	Reference
FTO/SnO_2/KCl/FA-perovskite/spiro-OMeTAD/Au	Tuning the CBD SnO_2	Anti-solvent	0.09370	25.4 (certified 25.2%)	S1
FTO/SnO_2/KFAMDA-perovskite/MeO-PEAI/spiro-OMeTAD/Au	Coherent interlayer ($FASnCl_x$)	Anti-solvent	0.09540	25.8 (certified 25.5%)	S2
FTO/c-TiO_2/m-TiO_2/$FAPbI_3$/OAI/spiro-OMeTAD/Au	Ion-modulated radical doping	Anti-solvent	0.0803	25.1 (certified 25.0%)	S3
FTO/SnO_2/$FAPbI_3$/PEAI/spiro-OMeTAD/Au	Inactive $(PbI_2)_2RbCl$	Two-step	0.07414	26.1 (certified 25.6%)	36
ITO/SnO_2/FAMACs-perovskite/PPEAI/spiro-OMeTAD/Au	Rb-based perovskitoid scaffold	Anti-solvent	0.0706	25.14	15
ITO/SnO_2/$FA_xMA_{1-x}PbI_3$/OACl/spiro-OMeTAD/Au	Pre-annealing treatment	Two-step	0.058424	24.95 (certified 24.17%)	S4
FTO/c-TiO_2/SnO_2/$Cs_{0.05}MA_{0.05}FA_{0.9}PbI_3$/PEAI/spiro-OMeTAD/Au	Pentanamidine hydrochloride (PAD) additive	Vacuum flash	0.0550	25.4 (certified 25.0%)	S5
FTO/SnO_2/KCl/$FAPbI_3$/spiro-OMeTAD/Au	Volatile alkylammonium chlorides	Anti-solvent	0.09597	26.08 (certified 25.73%)	S6
FTO/SnO_2/$FAPbI_3$/Phenylethanamine salt/spiro-OMeTAD/Au	Anion–π interactions	Two-step	0.08313	26.07 (certified 25.8%)	S7
ITO/$SrSnO_3$/$Cs_{0.05}(FAMA)_{0.95}PbI_3$/OATsO/spiro-OMeTAD/Au	$SrSnO_3$ electron transport layer	Two-step	0.049	25.17	S8
PI/ITO/SnO_2/FAMACs-perovskite/PPAI/spiro-OMeTAD/Au	Interfacial modification via PF	Anti-solvent	0.0601	24.61 (certified 23.51%)	S9
ITO/SnO_2/FAMACs-perovskite/spiro-OMeTAD/Au	All-interface defect passivation	Two-step	0.1196	25.43 (certified 24.4%)	S10
FTO/SnO_2/KCl/FAMACsRb-perovskite/PEAI/spiro-OMeTAD/MoO_3/Ag	Dibromosuccinic acid (DBSA) additive	Anti-solvent	0.09	25.41 (certified 25.0%)	S11
FTO/SnO_2/$FAPbI_3$/PEAI/spiro-OMeTAD/MoO_3/Ag	IP-TFSI initiator	Two-step	0.0582	25.16 (certified 24.85%)	S12

FTO/c-TiO_2/SnO_2/$FAPbI_3$/ $H_2C_2O_4$/spiro-OMeTAD/Au	Lead oxalate (PbC_2O_4) compact layer	Anti-solvent	0.0676	25.39 (certified 24.92%)	S13
FTO/SnO_2/RbFAMA-perovskite/PEAI/spiro-OMeTAD/Au	SnO_2/$MDACl_2$ Interlayer	Two-step	0.09	25.28	18
FTO/SnO_2/KCl/RbFAMA-perovskite/2-Cl-PEAI/spiro-OMeTAD/Au	Interfacial modification via AEP	Two-step	0.10126	26.40 (certified 25.98%)	S14
FTO/SnO_2/$Cs_{0.05}MA_{0.05}FA_{0.9}PbI_3$/A6BfPI/spiro-OMeTAD/Au	2D perovskitoid $(A6BfP)_8Pb_7I_{22}$ passivation layer	Anti-solvent	0.0625	26.1 (certified 24.6% for 1.04 cm^2)	S15
FTO/SnO_2/CsFAMA-perovskite/2-Cl-PEAI/T2/Au	T2 hole-transporting material	Thermal evaporation	0.09973	26.41 (certified 26.21%)	S16
FTO/c-TiO_x/m-TiO_x/$FAPbI_3$/CMAI/spiro-OMeTAD/Au	Interfacial modification via Li_2CO_3 and BAE	Anti-solvent	0.0741	26.5 (certified 26.31%)	S17
FTO/SnO_2/RbFAMA-perovskite/passivation layer/spiro-OMeTAD/Au	Binary passivation (PPAI and tBBAI)	Two-step	0.07461	26.75 (certified 26.0%)	S18
FTO/SnO_2/KCl/FAMAMDA-perovskite/passivation/spiro-OMeTAD/Au	Excess ligands for CBD SnO_2	Anti-solvent	0.09567	26.4 (certified 26.1%)	S19
FTO/SnO_2/KCl/ $FAPbI_3$/4-MeO-PEAI/spiro-OMeTAD/Au	Non-invasive surface reaction via 4-MeO-PEAI	Anti-solvent	0.085	26.13 (certified 25.66%)	S20
FTO/SnO_2/RbFAMA-perovskite/PEAI/spiro-OMeTAD/Au	$AlCl_3$ interlayer	Two-step	0.0660	26.54% (certified 26.29%)	This work

Table S7. Performance data for the large-area control and target PSCs.

Condition	V_{oc} (V)	J_{sc} (mA cm^{-2})	FF	PCE (%)
control, reverse scan	1.168	26.15	0.790	24.14
control, forward scan	1.144	26.15	0.768	22.97
target, reverse scan	1.196	26.34	0.807	25.44
target, forward scan	1.186	26.35	0.791	24.72

Table S8. EIS parameters of control and target PSCs.

Condition	R_s (Ω cm^2)	C_1 (F cm^{-2})	R_{tr} (Ω cm^2)	C_2 (F cm^{-2})	R_{rec} (Ω cm^2)
Control	1.00	6.87E-8	6495.0	6.52E-6	1.14E5
Target	0.86	6.39E-8	4325.0	4.65E-6	1.15E6

Table S9. Photovoltaic parameters of the control cell under different light intensities.

Light intensity (mW cm^{-2})	V_{oc} (V)	J_{sc} (mA cm^{-2})	FF	PCE (%)
100	1.170	26.13	0.804	24.59
90	1.167	24.57	0.785	22.52
80	1.161	20.80	0.784	18.93
70	1.158	18.08	0.771	16.15
60	1.153	15.56	0.786	14.09
50	1.144	13.64	0.788	12.30
40	1.132	10.28	0.788	9.16
30	1.122	7.96	0.782	6.99
20	1.105	5.41	0.777	4.64
10	1.070	2.62	0.742	2.08

Table S10. Photovoltaic parameters of the target cell under different light intensities.

Light intensity (mW cm^{-2})	V_{oc} (V)	J_{sc} (mA cm^{-2})	FF	PCE (%)
100	1.197	26.37	0.826	26.09
90	1.195	24.70	0.819	24.19
80	1.192	21.04	0.828	20.77
70	1.189	18.14	0.826	17.81
60	1.183	15.71	0.832	15.46
50	1.179	13.71	0.827	13.38
40	1.171	10.41	0.825	10.06
30	1.163	8.03	0.834	7.79
20	1.149	5.50	0.837	5.29
10	1.123	2.64	0.831	2.46

Table S11. Performance data for the control cell before and after encapsulation.

Condition	V_{oc} (V)	J_{sc} (mA cm^{-2})	FF	PCE (%)
pristine, reverse scan	1.150	26.06	0.783	23.47
pristine, forward scan	1.127	26.13	0.752	22.15
encapsulated, reverse scan	1.138	24.22	0.730	20.11
encapsulated, forward scan	1.124	24.15	0.717	19.46

Table S12. Performance data for the target cell with and without a shadow mask during measurement.

Condition	V_{oc} (V)	J_{sc} (mA cm^{-2})	FF	PCE (%)
w/o mask, reverse scan	1.191	26.21	0.822	25.65
w/o mask, forward scan	1.189	26.22	0.813	25.37
w/ mask, reverse scan	1.166	26.18	0.837	25.54
w/ mask, forward scan	1.162	26.18	0.831	25.27

References

[S1] J. J. Yoo, G. Seo, M. R. Chua, T. G. Park, Y. Lu, F. Rotermund, Y.-K. Kim, C. S. Moon, N. J. Jeon, J.-P. Correa-Baena, V. Bulović, S. S. Shin, M. G. Bawendi, J. Seo, *Nature* **2021**, *590*, 587.

[S2] H. Min, D. Y. Lee, J. Kim, G. Kim, K. S. Lee, J. Kim, M. J. Paik, Y. K. Kim, K. S. Kim, M. G. Kim, T. J. Shin, S. I. Seok, *Nature* **2021**, *598*, 444.

[S3] T. Zhang, F. Wang, H.-B. Kim, I.W. Choi, C. Wang, E. Cho, R. Konefal, Y. Puttisong, K. Terado, L. Kobera, M. Chen, M. Yang, S. Bai, B. Yang, J. Suo, S.-C. Yang, X. Liu, F. Fu, H. Yoshida, W. M. Chen, J. Brus, V. Coropceanu, A. Hagfeldt, J.-L. Brédas, M. Fahlman, D. S. Kim, Z. Hu, F. Gao, *Science* **2022**, *377*, 495.

[S4] H. Wang, F. Ye, J. Liang, Y. Liu, X. Hu, S. Zhou, C. Chen, W. Ke, C. Tao, G. Fang, *Joule* **2022**, *6*, 2869.

[S5] P. Shi, Y. Ding, B. Ding, Q. Xing, T. Kodalle, C. M. Sutter-Fella, I. Yavuz, C. Yao, W. Fan, J. Xu, Y. Tian, D. Gu, K. Zhao, S. Tan, X. Zhang, L. Yao, P. J. Dyson, J. L. Slack, D. Yang, J. Xue, M. K. Nazeeruddin, Y. Yang, R. Wang, *Nature* **2023**, *620*, 323.

[S6] J. Park, J. Kim, H.-S. Yun, M. J. Paik, E. Noh, H. J. Mun, M. G. Kim, T. J. Shin, S. I. Seok, *Nature* **2023**, *616*, 724.

[S7] Z. Huang, B. Yang, X. Huang, J.-T. Li, Y. Wu, Y. Chen, K. Li, X. Niu, N. Li, G. Liu, Z. Yu, H. Zai, Q. Chen, T. Lei, L. Wang, H. Zhou, *Nature* **2023**, *623*, 531.

[S8] C. Luo, G. Zheng, F. Gao, X. Wang, C. Zhan, X. Gao, Q. Zhao, *Nat. Photonics* **2023**, *17*, 856.

[S9] R. Xu, F. Pan, J. Chen, J. Li, Y. Yang, Y. Sun, X. Zhu, P. Li, X. Cao, J. Xi, J. Xu, F. Yuan, J. Dai, C. Zuo, L. Ding, H. Dong, A. K. -Y. Jen, Z. Wu, *Adv. Mater.* **2023**, *36*, 2308039.

[S10] L. Shen, P. Song, L. Zheng, L. Wang, X. Zhang, K. Liu, Y. Liang, W. Tian, Y. Luo, J. Qiu, C. Tian, L. Xie, Z. Wei, *Adv. Mater.* **2023**, *35*, 2301624.

[S11] Q. Wang, Y. Chen, X. Chen, W. Tang, W. Qiu, X. Xu, Y. Wu, Q. Peng, *Adv. Mater.* **2023**, *36*, 2307709.

[S12] H. Yang, T. Xu, W. Chen, Y. Wu, X. Guo, Y. Shen, C. Ding, X. Chen, H. Chen, J. Ding, X. Wu, G. Zeng, Z. Zhang, Y. Li, Y. Li, *Angew. Chem. Int. Ed.* **2023**, *63*, e202316183.

[S13] T. Chen, J. Xie, B. Wen, Q. Yin, R. Lin, S. Zhu, P. Gao, *Nat. Commun.* **2023**, *14*, 6125.

[S14] M. Li, B. Jiao, Y. Peng, J. Zhou, L. Tan, N. Ren, Y. Ye, Y. Liu, Y. Yang, Y. Chen, L. Ding, C. Yi, *Adv. Mater.* **2024**, *36*, 2406532.

[S15] C. Liu, Y. Yang, H. Chen, I. Spanopoulos, A. S. R. Bati, I. W. Gilley, J. Chen, A. Maxwell, B. Vishal, R. P. Reynolds, T. E. Wiggins, Z. Wang, C. Huang, J. Fletcher, Y. Liu, L. X. Chen, S. D. Wolf, B. Chen, D. Zheng, T. J. Marks, A. Facchetti, E. H. Sargent, M. G. Kanatzidis, *Nature* **2024**, *633*, 359.

[S16] J. Zhou, L. Tan, Y. Liu, H. Li, X. Liu, M. Li, S. Wang, Y. Zhang, C. Jiang, R. Hua, W. Tress, S. Meloni, C. Yi, *Joule* **2024**, *8*, 1691.

[S17] Q. Li, H. Liu, C.-H. Hou, H. Yan, S. Li, P. Chen, H. Xu, W.-Y. Yu, Y. Zhao, Y. Sui, Q. Zhong, Y. Ji, J.-J. Shyue, S. Jia, B. Yang, P. Tang, Q. Gong, L. Zhao, R.

Zhu, *Nat. Energy* **2024**, *9*, 1506.
[S18] Z. Qu, Y. Zhao, F. Ma, L. Mei, X.-K. Chen, H. Zhou, X. Chu, Y. Yang, Q. Jiang, X. Zhang, J. You, *Nat. Commun.* **2024**, *15*, 8620.
[S19] G. Seo, J. J. Yoo, S. Nam, D. S. Lee, S. Gao, B. K. Kim, S. J. Sung, B. J. Kang, D. W. deQuilettes, J. Park, J.-S. Park, I. S. Cho, F. Rotermund, S. I. Seok, S. S. Shin, *Nat. Energy* **2025**, *10*, 774.
[S20] K. Zhang, Y. Wang, L. Guo, M. Tao, H. Guo, X. Zhang, Z. Song, J. Wen, Y. Yang, J. Shao, H. Zhou, Y. Song, *Nat. Synth.* **2025**, DOI https://doi.org/10.1038/s44160-025-00865-w.
